# The specific linear or curved boundaries between WHO grade II–III insular gliomas and the basal ganglia indicate distinct biological features, survival outcomes, and surgical strategies: evidence from 330 cases

**DOI:** 10.1016/j.nicl.2026.103995

**Published:** 2026-04-25

**Authors:** Hongfang Zhao, Bohan Zhang, Bowen Xue, Jianfeng Liang, Changyu Lu, Zonggang Hou, Zhenye Li, Jian Xie

**Affiliations:** a Capital Medical University, Beijing, China; b Department of Neurosurgery, Beijing Tiantan Hospital, Capital Medical University, Beijing, China; c Department of Neurology, Beijing Tiantan Hospital, Capital Medical University, Beijing, China; d Department of Neurosurgery, Peking University International Hospital, Beijing, China

**Keywords:** Insular glioma, WHO grade II–III, Boundary shape, Morphology, Prediction value

## Abstract

**Background:**

WHO grade II–III insular glioma (InG) can displace the basal ganglia, resulting in distinct linear or curved boundary shapes. This study retrospectively explored this morphological distinction and its clinical relevance.

**Methods:**

MRI and clinical data were collected from Beijing Tiantan Hospital over the past 20 years. The boundary shape on the FLAIR sequence was characterized using the fractal dimension (FD) and curved parameters. Statistical analyses included the χ^2^ test, Cox regression, Kaplan–Meier analysis, and correlation analysis. Regression models were constructed using the stepwise Wald method. Model performance was evaluated through internal validation, including bootstrap resampling and 5-fold cross-validation.

**Results:**

A total of 330 patients were included. Based on the FD, patients were classified into linear ('L'; 49%) and curved ('C'; 51%) subgroups. Compared with the L subgroup, the C subgroup exhibited more favorable biological features and progression-free survival (PFS) (*p* < 0.001). Using routine pathological variables, regression models were constructed to identify crucial factors in different subgroups and perform disease stratification for Statistical explanation. Internal validation supported model stability. With regard to surgical responses, although the rate of gross total resection (GTR) was higher in the L subgroup, the PFS was not increased, whereas that of the C subgroup demonstrated an increase (*p* < 0.001).

**Conclusions:**

The boundary shape between WHO grade II–III InG and the basal ganglia may reflect differences in biological features, survival outcomes, and surgical responses. Thus, the boundary shape should be considered in clinical practice.

## Highlights

• The boundary shape of insular glioma could provide clinical suggestions.
• It indicated different tumor features, survivals and surgical strategies.
• The benefits of surgery may vary depending on the boundary shape.

## 1 Background

As a common intracranial malignant tumor, glioma severely affects patients’ lives (Shi et al., 2022). There have been many advances in technology regarding imaging methods, especially with the development of high-resolution multimodal MRI. This approach is used by clinicians to evaluate glioma features and prognoses based on specific characteristics, such as tumor enhancement and the mismatch sign (Abrigo et al., 2018, Patel et al., 2017, Choi et al., 2011). Clinical evidence has demonstrated that the insula is frequently involved in supratentorial low-grade gliomas (Li et al., 2023). Approximately 90% of insular gliomas (InGs) are diagnosed as grades II–III (Li et al., 2020, Sanai et al., 2010). They exhibit similar MRI characteristics, such as clear demarcation from normal tissue and performance on different imaging sequences. Thus, traditional MRI cannot effectively distinguish differences in biological behaviors or growth trends of InGs. In addition, it is limited in terms of providing valuable information for clinicians to assess prognoses and surgical responses.

Previous radiomic studies have identified several promising novel parameters and their combinations with potential clinical applications (Hui et al., 2016, Xue et al., 2025, Drexler et al., 2025, Lambin et al., 2012, Cepeda et al., 2021, Yang et al., 2016). To explore more direct indicators, Pérez-Beteta et al. (2018) analyzed 216 glioblastoma (GM) MR images and revealed that tumor surface regularity may reflect the tumor growth process, survival outcomes and surgical responses (Pérez-Beteta et al., 2018). Subsequently, a strong association has been confirmed between tumor surface regularity and the characteristics of other malignant tumors (Morioka et al., 2019). Given the limitations of this parameter for evaluating grade II–III InGs (i.e., lower grade), more individualized indicators should be explored. Regarding the insular anatomical structure, InG seems to exhibit either a curved or linear growth pattern, with its inner edges pushing or spreading inward, resulting in distinct boundary patterns (Varnavas and Grand, 1999). However, few studies have examined specific boundary shapes and their biological mechanism and clinical value are yet unclear.

In this study, we analyzed the different boundary shapes between WHO II–III InG and the basal ganglia, investigated their biological features using routine clinicopathological variables, and compared patient survival and surgical responses across the boundary shapes. The study findings can provide suggestions for medical care and surgical strategies in the future.

## 2 Methods

### 2.1 Patients

#### 2.1.1 Inclusion criteria

**Fig. 1:**
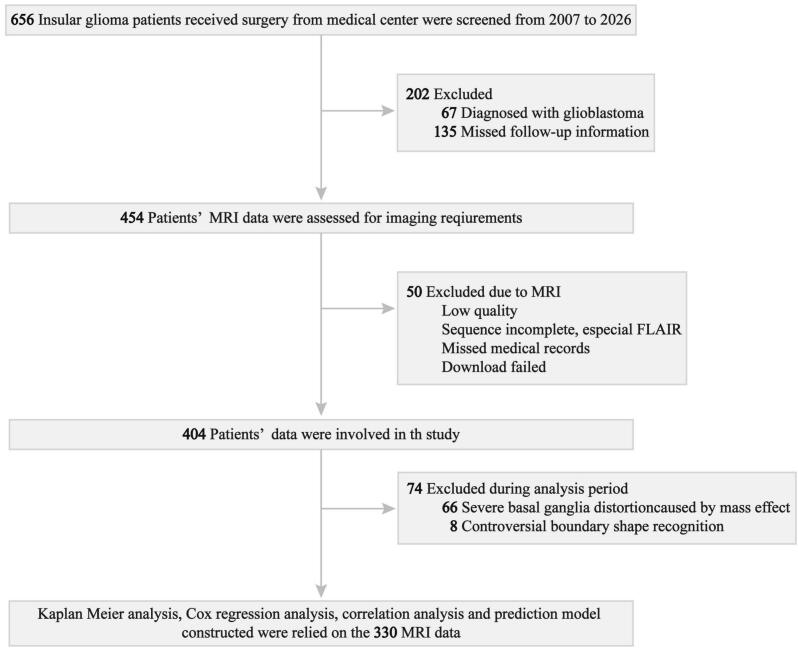
Flowchart of patient inclusion in this study.

This study was a retrospective analysis and received approval from the Beijing Tiantan Hospital. The requirement for written informed consent was waived because of the retrospective nature of the study. This study was reported in accordance with the STROCSS guidelines (Agha et al., 2025). Clinical information over the past two decades was collected from a single medical records room of the Beijing Tiantan Hospital. The pathological diagnosis was confirmed according to the World Health Organization Classification of Tumors of the Central Nervous System. Up to June 2025, 656 patients with InG were enrolled consecutively. A total of 276 patients were excluded because of missing follow-up information (n = 135), presence of WHO grade IV tumor (n = 67), severe basal ganglia distortion/invasion (n = 66), or controversial boundary shape (n = 8). Finally, 330 patients were included in this study (Fig. 1). The detailed inclusion criteria were as follows: i) unifocal InG with a clear boundary between the tumor and the basal ganglia; ii) mild mass effect that did not result in severe basal ganglia distortion; and iii) available data on clinical variables, including age, gender, laterality, tumor volume, routine pathological results during treatment, extent of resection (EOR, assessed via T1c imaging within 72 h after surgery), and survival status (recorded from the date of preoperative MRI to the last follow-up or tumor recurrence, as confirmed by imaging).

#### 2.1.2 Routine pathology assessment and interpretation standards

The histopathological information obtained from medical records was confirmed through a consistent process. As a part of the routine clinical workflow following standard procedures, the assessments were performed by the pathology department of the Beijing Tiantan Hospital. In brief, histological classification was performed via hematoxylin–eosin staining. The statuses of IDH1, ATRX, TP53, and Ki–67 were confirmed via immunohistochemistry (IHC). The 1p/19q co-deletion and MGMT methylation statuses were determined via fluorescence in situ hybridization (FISH) and pyrosequencing, respectively. The experimental details are summarized in Table S1. All pathological interpretations were independently performed by two certified neuropathologists. The interpretation standards were as follows:

With regard to IHC staining, tumor cells were considered to possess IDH1 mutations if they demonstrated strong diffuse cytoplasmic immunoreactions with the IDH1 antibodies. Few samples exhibited strong but focal staining reactions, with a positive threshold of ≥ 30% immunoreactive tumor cells. Similarly, when ATRX antibodies were used, if tumor cell nuclei were not stained similar to non-neoplastic cells, they were considered to possess ATRX mutations. If the tumor cell nuclei were stained when TP53 antibodies were used, they were considered to possess TP53 mutations. The positive thresholds of ATRX and TP53 mutations were ≥ 10% of stained tumor nuclei. When Ki–67 antibodies were used, the Ki–67 index was calculated as the proportion of stained tumor nuclei among the total tumor nuclei in proliferative areas under 400 × magnification. With regard to the 1p/19q status, co-deletion was defined as ≥ 25% of the locus-specific fluorescent probe signals (from 100 nonoverlapping samples) exhibiting concurrent loss of both the 1p and 19q signals. With respect to the MGMT status, the cutoff value was ≥ 10% on the basis of the mean methylation level across the analyzed CpG sites (Capper et al., 2010, Reuss et al., 2015, Wiestler et al., 2013, Takami et al., 2015, Cai et al., 2014, Xie et al., 2015, Pandith et al., 2023).

#### 2.1.3 Image acquisition and preprocessing

MRI data were obtained using a 3.0 T scanner (GE Discovery MR750). The equipment performance is summarized in Table S2. Using SPM12 (version 7771, Wellcome Trust Centre for Neuroimaging, UK) in MATLAB (version R2022b, The MathWorks, US), two imaging experts (namely HFZ, with 4 years of experience in tumor segmentation and BHZ, with 3 years of experience) conducted preprocessing of the MRI data as follows: 1) data conversion (DICOM to NIfTI); 2) image reorientation (the standard AC–PC line); 3) registration: a. FLAIR to T1 registration, b. spatial normalization to the MNI space (template: ch2.nii); 4) resampling (1 cm3); and 5) bias field correction and intensity normalization (bias regularization: 0.001; bias FWHM: 60 mm). Tumor segmentation was conducted via two methods. First, preprocessed multimodal imaging sequences (FLAIR, T1, T2, and T1c) were imported into the U-Net_v2 deep learning model, which automatically conducted tumor segmentation. The model learning curves are shown in Fig. S1. Subsequently, the segmentation results were screened by HFZ and BHZ. Inaccurate masks were reanalyzed using the software ITK-SNAP (version 3.6.0; National Institutes of Health, US) in a semiautomatic manner. Finally, JX reviewed each tumor segmentation until accurate segmentation was identified.

**Fig. 2:**
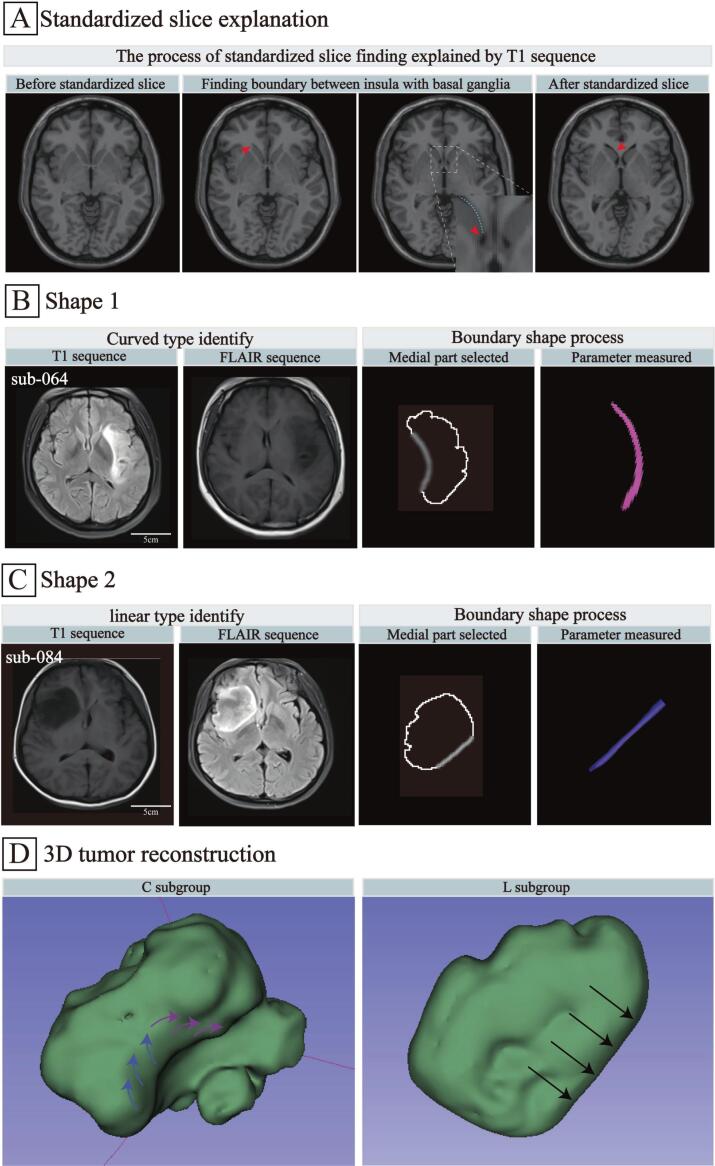
**Illustration of the standardized slice and specific boundary shapes on MRI. A:** Identification of the standardized slice on the axial T1-weighted sequence. During parameter measurement, the operator gradually scrolled from the cranial to the caudal direction, until the bilateral frontal horns of the lateral ventricles first appeared. After careful reviewed, the slice at which the lateral ventricles were fully visible or the genu of the corpus callosum was clearly identified was defined as the standardized slice. **B:** Determination and processing of the curved shape; **C:** Determination and processing of the linear shape; **D:** Three-dimensional tumor reconstruction. The left image shown the curved shape's spreading along the basal ganglia rather than squeezing deep structures. The right picture exhibited the linear shape's extruded the basal ganglia causing structure distortion. **Abbreviations:** A: Above; S: Sub; R: Rear; I: Inside.

For boundary shape determination, the axial FLAIR slice exhibiting 'rostrum of corpus callosum-corpus callosum' clearly was considered the standard reference. Additionally, the contour of the frontal horns of the lateral ventricles should appear initially in this slice. It was regarded as another method to identify the criterion slice accurately (Fig. 2A). Notably, in some cases where InG caused subtle changes in the position of the basal ganglia, identifying the standard slice may be difficult. In this scenario, we scrolled through adjacent planes carefully and combined this process with grayscale threshold adjustment to ensure the accurate identification of the boundary.

#### 2.1.4 Geometric measures

In the specific slice, the FD of the boundary shape between WHO grade II–III InG and the basal ganglia was measured four times using the semiautomatic system of the FracLac plugin (version 2015Sep090313a9330; Charles Sturt University, Australia) in ImageJ (version 1.53; National Institutes of Health, Bethesda). Subsequently, the mean values were calculated by HFZ and BHZ to objectively determine the boundary shape (Fig. 2B–C). Furthermore, if the boundary shape was curved, five additional parameters, namely the total curvature, maximum curvature, average curvature, curvature variation and tortuosity, were calculated using the kappa plugin (version 2.0.0; McGill University, Canada). The calculation formulas were as follows:

(1) $$\text{FD}=\underset{\varepsilon \text{→0}}{\text{lim}}\frac{\text{log}\text{N}(\varepsilon )}{\text{log}\text{1}/\varepsilon }$$

(2) $$\text{Total curvature}=\int \left|\text{k}(\text{s})\right|\text{ds}$$

(3) $$\text{Maximum curvature}=\text{max}\;\left|\text{k}(\text{s})\right|$$

(4) $$\text{Average curvature}=\frac{\text{1}}{\text{2}}\;\text{(}{\text{k}}_{\text{1}}+{\text{k}}_{\text{2}})$$

(5) $$\text{Curvature variation}=\frac{\text{d}\kappa }{\text{ds}}$$

(6) $$\text{Tortuosity}=\frac{\text{L}}{\text{D}}$$

A detailed explanation of the formulas is provided in the Supplementary methods protocol. Three-dimensional tumor construction was performed to determine the different boundary shapes (Fig. 2D).

## 3 Statistical methods

Statistical analyses were performed using GraphPad Prism (version 8.0.2; GraphPad Software, US), Python (version 3.10; Python Software Foundation, USA), and PyCharm (version 2025.1; JetBrains, Czech Republic). The relationships between the boundary shape and clinical categorical variables were assessed by the χ2 test. The associations between the variables were explored using regression analyses. The relationships between variable pairs were assessed by calculating the Spearman’s rank correlation coefficient. Multicollinearity among covariates was assessed based on the variance inflation factor (VIF) (Marquardt, 1970). The variables exhibiting both a high correlation and a high VIF were excluded from analyses (coefficient > 0.7, *p* < 0.05 and VIF > 10). Survival between the groups was compared using Kaplan‒Meier analysis and Mantel‒Cox and Breslow‒Wilcoxon tests. Finally, regression models were constructed using the stepwise Wald method. Robustness of the models was confirmed through internal validation, including bootstrap resampling and 5-fold cross-validation. The significance threshold was *p* < 0.05 (two–tailed). The relevant environments and packages are summarized in Table S3.

## 4 Results

### 4.1 The specific boundary shape reflected different biological features of InG

**Fig. 3:**
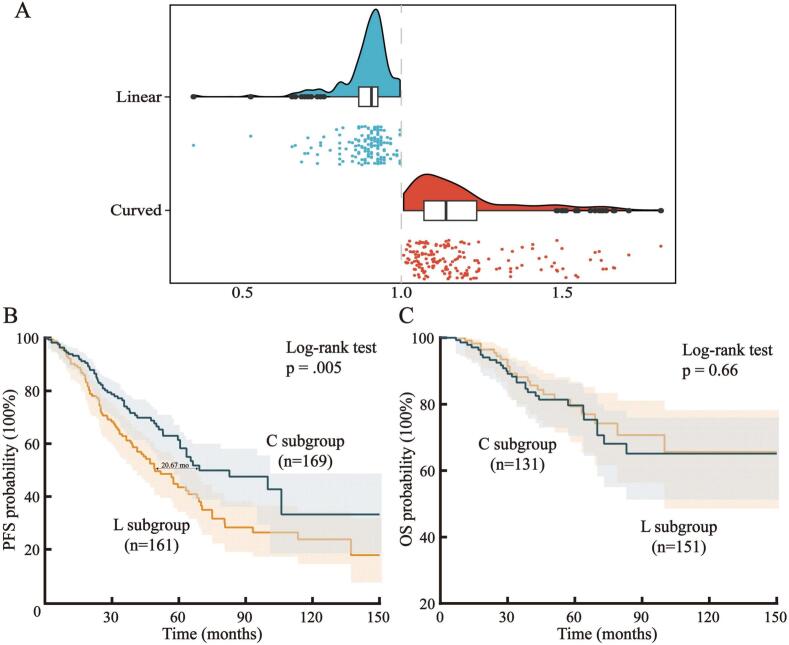
**Results of FracLac measurements and Kaplan**–**Meier analyses. A:** Boundary shapes could be classified into two subgroups (linear and curved, labeled as 'L' and 'C') by fractal dimension; **B:** Kaplan–Meier analysis of PFS for the C and L subgroups; **C:** Kaplan–Meier analysis of OS for the C and L subgroups. **Abbreviations:** C: curved; L: linear; PFS: progression-free survival; OS: overall survival; mo: months; *p*: p value.

Initially, we discovered that the boundary shape was influenced by the main body of the tumor. On the one hand, some InGs exhibited sharp or abrupt angles because of potential tumor heterogeneity. On the other hand, gliomas could also exhibit nearly mirror-like curved medial inner regions because of their origin from the anterior and posterior insular regions (Fig. S2). However, the measured FD was not affected in the latter exceptional cases. Finally, depending on boundary shape, we classified patients into linear and curved subgroups (labeled as 'L' and 'C') on the basis of whether the FD was ≤ 1 or > 1 and conducted further analyses (Fig. 3A) (Di Ieva et al., 2024).

Univariate regression analysis included routine pathological variables and revealed that compared with those in the L subgroup, the proportions of tumors that were oligodendroglioma (OGS), and exhibited 1p/19q co-deletion, ATRX mutation, TP53 mutation, and a Ki–67 index of ≤ 10% were relatively higher in the C subgroup. Moreover, among patients aged ≥ 38 years, a curved boundary shape was more common. The detailed information is summarized in Table 1. Multivariate regression analysis revealed that tumors with a C shape exhibited relatively stable biological features, such as having a WHO grade II, having IDH1 mutation, being an OGS, and having a Ki–67 index of ≤ 10%. No significant correlation was found between variable pairs. Despite the significant difference in laterality between two subgroups, we considered the result to be dissociated from clinical practice (Tables S4–S7).

**Table 1:** Summary of clinical characteristics and univariate analyses of boundary shape in 330 patients with WHO grade II–III insular glioma.

**Variables**		**Subgroup**			
		**Linear**	**Curved**	***p***	**OR**
Age (years)	≤38	91	58	<0.001	0.40 (0.26–0.63)
	>38	70	111		
Gender	Male	85	92	0.83	0.94 (0.60–1.45)
	Female	76	77		
Side	Left	78	89	0.51	0.84 (0.54–1.30)
	Right	83	80		
Volume (cm^3^)	≤20.17	22	12	0.07	2.07 (0.98–4.27)
	>20.17	139	157		
Histological classification	OGS	46	71	0.03	0.57 (0.35–0.95)
	Non-OGS	74	65		
WHO grade	2	108	117	0.72	0.91 (0.58–1.43)
	3	53	52		
IDH1 status	Mutant	73	85	0.84	0.92 (0.44–1.98)
	Wild	14	15		
1p/19q status	co-delete	31	49	0.05	0.55 (0.32–0.97)
	intact	72	63		
	IDH**^+^** with 1p/19q status	9	44	0.02	0.39 (0.16–0.87)
	IDH**^+^** without 1p/19q status	34	65		
ATRX status	Mutant	59	49	0.04	0.57 (0.33–0.96)
	Wild	43	63		
MGMT status	Methylated	52	53	0.68	0.81 (0.37–1.76)
	Unmethylated	17	14		
TP53	Mutant	71	58	0.02	0.54 (0.33–0.86)
	Wild	58	88		
Ki–67 index	≤10%	38	64	0.04	0.57 (0.34–0.96)
	>10%	70	67		
Epilepsy	Yes	95	95	0.66	1.21 (0.72–1.72)
	No	66	74		

**Abbreviations:** OR: Odds ratio; WHO: World Health Organization; OGS: Oligodendroglioma; IDH1: Isocitrate dehydrogenase 1; 1p/19q: chromosomal arms 1p and 19q; MGMT: O_6_-methylguanine-DNA methyltransferase; ATRX: Alpha-thalassemia/mental retardation syndrome X-linked; TP53: Tumor protein p53; Ki–67: Ki–67 labeling index; IDH1^+^: IDH1 mutation; *p*: p value.

### 4.2 The specific boundary shape reflected different survival outcomes for InG

Regarding survival outcomes, we found that the recurrence rate of tumors in the L subgroup was higher than that of tumors in the C subgroup (53.4% vs. 36.7%, *p* < 0.001; Fig. S3A). The Kaplan–Meier analysis supported these results and also revealed that the PFS of patients in the L subgroup was shorter than that of patients in the C subgroup, with a median survival difference of 20.67 months. No difference was observed in the overall survival (OS) between the groups (Fig. 3B–C).

To obtain further statistical inference, we constructed regression models for each boundary shape using the stepwise Wald method; these models were used to identify important factors and perform disease stratification. To ensure accurate clinical explanation, all routine variables were considered in the analysis. The continuous variables were divided on the basis of the data distribution features and clinical values (Table S8) (Cai et al., 2014, Weller et al., 2021). After the exclusion basing correlation coefficient and VIF, variables with a *p* value of < 0.05 were included. The corresponding coefficients were used as the weights of the variables in the model. Finally, the analysis revealed the best extended Cox regression model for the L subgroup, which had five variables and the related coefficients (Tables S9–12). The model is expressed by the following equation:

Risk_L = (0.78)*Ki–67 status + (0.54)*epilepsy status + (−0.77)*TP53 status + (−0.90)*1p/19q status + (0.55)*tumor volume status

Based on the points scale, the best threshold was 23, which could divide the patients in the L subgroup into high- and low-risk cohorts (Table S13). A significant difference was noted in PFS between the two cohorts (log–rank *p* = 0.003; Fig. S4A). Bootstrap resampling revealed model stability (C index = 0.67). Moreover, 5-fold cross-validation initially supported its clinical value in prognosis assessment (mean C index = 0.63, ICI = 0.008, E50 = 0.006).

As for C subgroup, the analyses suggested that there were significant differences in the TC and tortuosity between patients with and without recurrence (Fig. S4B). As they were important parameters, we included them in model construction (Table S8). After the exclusion basing correlation coefficient and VIF, the best extended Cox regression model for tumors in the C subgroup included six factors and their corresponding coefficients (Tables S14–17). The regression model is given by the following equation:

Risk_C = (0.04)*Ki–67 status + (−0.86)*IDH1 status + (1.31)*age status +(1.39)*tumor volume status + (1.19)*Tortuosity status + (−1.70)*1p/19q status

Based on the points scale, the best threshold was 41 divided patients into high- and low- risk cohorts (Table S13). A significant difference was observed in PFS between patients in the two cohorts (log–rank *p* < 0.001; Fig. S4C). Internal validation supported model stability (C index = 0.84). In addition, 5-fold cross-validation initially demonstrated its value in the prognosis assessment (mean C index = 0.81, ICI = 0.061; E50 = 0.021).

### 4.3 The specific boundary shape reflected different surgical responses for InG

**Fig. 4:**
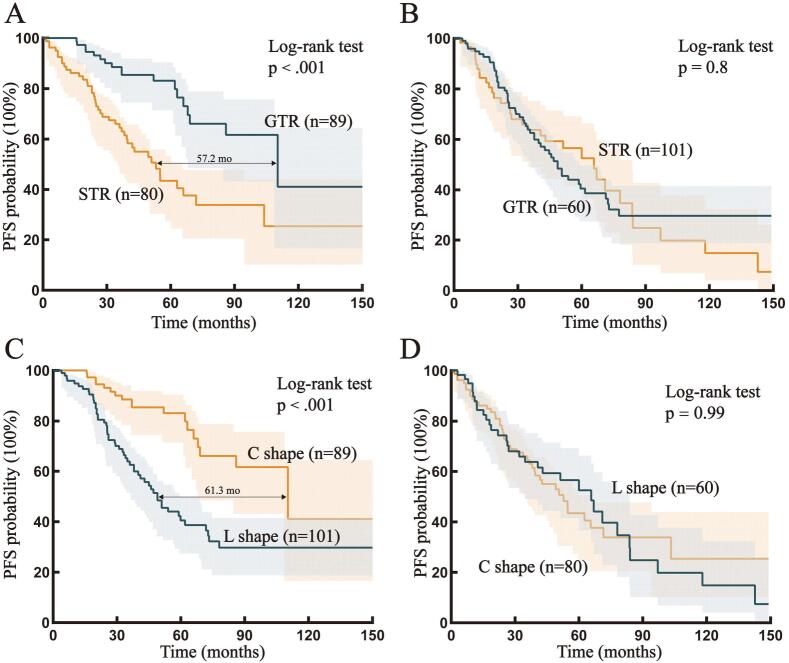
**Kaplan**–**Meier analyses of subgroups. A:** Kaplan–Meier analysis of PFS in GTR and STR patients within the C subgroup; **B:** Kaplan–Meier analysis of PFS in GTR and STR patients within the L subgroup; **C:** Kaplan–Meier analysis of PFS between the C and L subgroup within the GTR patients; **D:** Kaplan–Meier analysis of PFS between the C and L subgroup within the STR patients. **Abbreviations:** C: curved; L: linear; PFS: progression-free survival; OS: overall survival; mo: months; *p*: p value.

We compared surgical outcomes between patients in the two subgroups. The results demonstrated that the rate of gross total resection (GTR) was higher for tumors in the L subgroup than that for tumors in the C subgroup (53.16% vs. 42.25%; *p* = 0.04; Fig. S3B). Subsequently, we compared the PFS among patients in the different EOR groups in both the C and L subgroups. The Kaplan‒Meier analysis revealed that the GTR led to substantial improvements in the PFS among patients in the C subgroup (median PFS difference: 57.2 months; *p* < 0.001; Fig. 4A) compared with that among patients in the L subgroup (*p* = 0.8; Fig. 4B). For InGs with a curved boundary shape, the GTR resulted in improvements in the PFS (median PFS difference: 61.3 months; *p* < 0.001; Fig. 4C). However, for InGs that could only undergo subtotal resection because of multiple factors, the boundary shape did not have clinical value (*p* = 0.99; Fig. 4D).

Additionally, to determine whether the PFS of patients in different subgroups was influenced by the EOR, an additional multivariate regression analysis was performed for the GTR subgroup. The results revealed that the 1p/19q status, the TP53 status, the WHO grade, the Ki–67 index and the boundary shape (HR = 0.15, *p* < 0.001) were critical factors influencing disease progression (Tables S18–21). Thus, the clinical value of the boundary shape is not influenced by the EOR.

## 5 Discussion

Studies have reported that the insula is the most common region involved in supratentorial low-grade gliomas. Approximately 90% of these gliomas are diagnosed as WHO grade II or III (Pérez-Beteta et al., 2018, Su et al., 2019). Based on our experience, the boundary between WHO grade II–III InG and the basal ganglia is relatively defined and clear. Depending on the tumor location and inherent features, this boundary exhibits different shapes, particularly linear and curved shapes (Evleksiz et al., 2025). Considering the development of the insula and the basal ganglia, they originate from different germ layers and developmental regions, and involve distinct genetic landscapes. Despite some overlap between the blood supply and the fiber bundle system, there are several differences between these anatomical structures (Türe et al., 2000, Gogia et al., 2018, Nachtergaele et al., 2019). This may explain the non-interaction of the insula and the basal ganglia in some diseases (e.g., stroke and glioma) resulting in avoidance of the basal ganglia or a straight margin. To find novel description methods may provide perspectives to explore the mechanism and clinical value of the special imaging phenomenons (Abrigo et al., 2018, Hameed et al., 2018, Kerkhof and Vecht, 2013). Previous studies have reported that the FD can be used to objectively measure the geometric dimension and to capture subtle biological changes at the cellular, tissue, and organ levels (Mandelbrot, 1983). Smitha KA et al. (2015) reported that the FD increases with an increase in the grade of the glioma (Smitha et al., 2015). Chen C et al. (2018) revealed that the FD may be used as a prognostic indicator to predict the response of gliomas to chemotherapy (Chen et al., 2018). In our study, we divided patients with grade II–III InGs into linear and curved subgroups on the basis of the FD of the boundary. Analyses demonstrated that the different shapes may indicate distinct tumor growth processes, survival outcomes and surgery responses.

From a biological perspective, some routine pathological variables were significantly different between the two subgroups. The proportions of patients with grade II disease, IDH1 mutation, OGS, and a Ki–67 index ≤ 10% were higher in the C subgroup (*p* < 0.001). Combined with their clinical implications, we inferred that the biological behaviors were distinct in the two subgroups (Villanueva-Meyer et al., 2018, Fisher et al., 2002). It would assist clinicians to initially distinguish whether the InG characteristics was optimistic or not. We infer that relatively low-grade and/or slow-growing InGs may slowly spread along the natural anatomical structure of the basal ganglia, forming a smooth curved boundary. Conversely, relatively high-grade and/or proliferative InGs exhibit rapid growth, eventually compressing the normal structure of the basal ganglia and resulting in a relatively sharp linear boundary. Our results are supported by those of previous studies. Barras CD et al. (2009) reported that the shape of an intracerebral hemorrhage could reflect differences in disease stability and predict the growth rate of the hematoma (Barras et al., 2009). Philip NT et al. (2022) discovered that cerebral aneurysms exhibit various morphological features, such as spherical and pear shapes, and are associated with different risks of rupture because of distinct hemodynamic forces (Philip et al., 2022). Sánchez J et al. (2022) explored similar results for intracranial tumors and constructed a dynamic tumor interface model to explain the growth process of gliomas (Sánchez and Martín-Landrove, 2022). Pérez-Beteta et al. (2018) supported that a regular tumor surface was associated with a stable GM growth process (Pérez-Beteta et al., 2018). Meanwhile, this stable growth process has a relatively moderate influence on the normal structure of adjacent tissue (Sánchez and Martín-Landrove, 2022). Our study not only extended the scope of the current morphological findings but also identified personalized imaging indicators for InGs. The boundary shape may help clinicians further understand the biological features of InGs.

From a clinical perspective, although the overall survival did not significantly differ between the two subgroups, the PFS of patients in the C subgroup was higher than that of patients in the L subgroup (difference in median PFS > 20 months; p < 0.001). Thus, clinicians can initially assess the PFS on the basis of the boundary shape. Our analysis results are supported by some studies. Saad M et al. (2019) reported that irregular spherical tumors were associated with shorter survival in lung cancer patients (Saad et al., 2019). Huang Y et al. (2025) discovered a similar relationship for breast cancer (Huang et al., 2025). Pérez-Beteta et al. (2018) supported that tumor surface regularity was associated with the prognosis of GM (Pérez-Beteta et al., 2018). Compared with the existing indicators, our study provides an imaging indicator for a specific tumor area (Shahzadi et al., 2024, van Garderen et al., 2023). We constructed regression models for each boundary shape, described the influence of crucial variables on the PFS through statistical inference, and performed hierarchical assessment of the PFS for the L and C subgroups. The findings can help us further interpret how different parameters influence the distinct populations. The PFS-focused regression models were more meaningful for patients with InG, whose OS was inherently more favorable than that of patients in the other group. Additionally, internal validation supported the stability of the regression models. It cautiously meant that, with further validation in large-sized multiple cohorts, regression models incorporating the boundary shape may demonstrate potential assessment value in the future.

Owing to the potential influence of surgical outcomes on PFS and the risk of data leakage in the regression models, we conducted additional analyses of surgical outcomes between the two subgroups. Although the rate of GTR was higher among patients in the L subgroup (*p* < 0.001), their PFS was less favorable, whereas among patients in the C subgroup, the relationship was reversed (difference in mean PFS > 50 months; *p* < 0.001). Considering the effects of EOR, we performed additional analyses and found that the PFS of patients in the C subgroup was still more favorable in the GTR group (difference in mean PFS > 60 months, *p* < 0.001). In other words, the C subgroup would benefit from GTR. Based on the principle of maximizing tumor removal without compromising the patient's neurological function, we carefully suggest that tumors in the L subgroup had relatively high-grade and/or highly proliferative, which may result in a high recurrence risk (D'Amico et al., 2017, Hervey-Jumper and Berger, 2016). Thus, neurosurgeons need not repeatedly strive to observe the bean dreg-like (i.e., ricotta cheese) appearance of the normal basal ganglia (Xie et al., 2011). Although the aforementioned surgical concept can ensure adequate resection depth and achieve successful GTR, it may result in increased risks of neurological function damage. Moreover, benefits to the control of tumor recurrence and patient PFS was limited. If InGs have a curved boundary shape and spread along the basal ganglia implying relatively low grade and/or slow growth, surgeons should implement multiple surgical and monitoring techniques and pursue tumor resection until the basal ganglia is observed, which could greatly increase the PFS of the patient (Przybylowski et al., 2021, Michaud and Duffau, 2016, Liu et al., 2025). Our findings provide suggestions for making intraoperative decisions to balance between tumor resection and neurological function preservation. It would promote the neurosurgical strategy implementation that maximizing tumor removal while protecting the patient's neurological functions.

From a future perspective, more direct studies should be conducted in the future incorporating topological and morphometric mapping to explore the underlying mechanisms of different boundary shapes. Our previous studies provided suggestions for further exploration. For instance, iron content in the basal ganglia present in the interior is associated with glioma differentiation (Yang et al., 2024). The molecular status of InGs could influence the connectivity of fiber tracts, such as anterior and superior thalamic radiations (Yang et al., 2024). Therefore, investigating whether iron content in the basal ganglia affects InG growth into the gray matter in the interior and whether the biological features of InG influence the connectivity of fiber tracts within the basal ganglia may help us better understand the biological processes of the distinct boundary shapes between the InG and the basal ganglia. Additionally, our study has some limitations. First, because it was not a longitudinal study, the biological growth process of InG could not be accurately characterized. Second, because the inclusion period spanned more than two decades, patients were classified according to the WHO criteria applicable at that time, which may have influenced the results. Moreover, the histological subtypes of the included patients were not further refined, which may cause some cases to be identified as 'not otherwise specified' potentially introducing research bias. Last but not the least, although internal validation supported model robustness, analysis with external validation datasets should be performed in the future.

## 6 Conclusion

The boundary shapes between the WHO grade II–III InG and the basal ganglia may initially indicate differences in the biological features, survival outcomes and surgical responses of the glioma. These findings provide insights for clinicians to further understand the growth patterns of InGs, construct survival evaluation models, and develop personalized surgical strategies. Additionally, additional direct investigations and validation are necessary to explore the mechanisms underlying different boundary shapes and their clinical value.

**Declarations**

**Ethics approval and consent to participate**

As a retrospective study, we need not obtain written informed consent from subjects (patients) in this study.

**Consent for publication**

This manuscript did not contain any sensitive personal information. All authors have seen and approved the manuscript.

**Availability of data and materials**

The datasets used and/or analysed during the current study are available from the corresponding author on reasonable request. The core could be found in GitHub (https://github.com/Hongfang-Zhao/insular_glioma_topology).

**Funding**

This study was supported by the National Natural Science Foundation of China (No. 82172028).

## CRediT authorship contribution statement

**Hongfang Zhao:** Writing – review & editing, Writing – original draft, Visualization, Validation, Software, Resources, Methodology, Investigation, Formal analysis, Data curation, Conceptualization. **Bohan Zhang:** Writing – review & editing, Writing – original draft. **Bowen Xue:** Software. **Jianfeng Liang:** Writing – review & editing. **Changyu Lu:** Writing – review & editing. **Zonggang Hou:** Writing – review & editing. **Zhenye Li:** Writing – review & editing. **Jian Xie:** Writing – review & editing, Visualization, Validation, Supervision, Software, Resources, Project administration, Methodology, Investigation, Funding acquisition, Formal analysis, Data curation, Conceptualization.

## Data Availability

Data will be made available on request.

## Appendix A Supplementary data

Supplementary Fig. S1 Learning curve of the model. The U-Net_v2 deep learning model was trained using the Medical Segmentation Decathlon (http://medicaldecathlon.com/). All data from Task01_BrainTumour were used for training with 5-fold cross-validation and 200 epochs. The best model was utilized to conduct tumor segmentation. The hardware environment was based on Ubuntu 22.04 (Canonical Ltd., UK) operating system, equipped with a 14-core CPU, 32GB RAM, and an NVIDIA RTX 4090 GPU (24GB VRAM). The software environment was built on Python (version 3.12, Python Software Foundation, USA), and PyTorch (version 2.3.0, PyTorch Foundation, USA) with CUDA (version 12.1, NVIDIA, USA) support. The nnUNet_v2 framework was installed within an Anaconda virtual environment.

Supplementary Fig. S2 Special cases in the fractal dimension measured process. A: The insular glioma did not advance uniformly inward, possibly due to tumor heterogeneity; B: the part of insular glioma did advanced inward, which may be caused by tumor heterogeneity; C: The insular glioma may occur in the anterior insular lobe; D: The insular glioma may locate in the posterior insular lobe.

Supplementary Fig. S3 Analysis of recurrence and extent of resection. A: Recurrence risk between the L and C subgroups; B: Proportion of GTR and STR in L and C subgroups. Abbreviations: L: linear; C: curved; GTR: gross total resection; STR: subtotal resection; *: *p* < 0.05; **: *p* < 0.01; ***: *p* < .001.

Supplementary Fig. S4 Disease stratification based on the statistical regression model. A: Kaplan–Meier analysis of PFS between the high- and low- risk cohorts within L subgroup; B: Analysis of related parameters about curved shape; C: Kaplan–Meier analysis of PFS between the high- and low- risk cohorts within C subgroup. Abbreviations: PFS: progression-free survival; L: linear; C: curved; *p*: p value; n: number.

## References

[b0005] Abrigo J.M., Fountain D.M., Provenzale J.M. (2018). Magnetic resonance perfusion for differentiating low-grade from high-grade gliomas at first presentation. Cochrane Database Syst. Rev..

[b0010] Agha R.A., Mathew G., Rashid R. (2025). Revised Strengthening thereporting of cohort, cross-sectional and case-control studies in surgery (STROCSS) guideline: an update for the age of ArtificialIntelligence. Premier J. Sci..

[b0015] Barras C.D., Tress B.M., Christensen S. (2009). Density and shape as CT predictors of intracerebral hemorrhage growth. Stroke.

[b0020] Cai J., Yang P., Zhang C. (2014). ATRX mRNA expression combined with IDH1/2 mutational status and Ki-67 expression refines the molecular classification of astrocytic tumors: evidence from the whole transcriptome sequencing of 169 samples samples. Oncotarget.

[b0025] Capper D., Weissert S., Balss J. (2010). Characterization of R132H mutation-specific IDH1 antibody binding in brain tumors. Brain Pathol..

[b0030] Cepeda S., Pérez-Nuñez A., García-García S. (2021). Predicting short-term survival after gross total or near total resection in glioblastomas by machine learning-based radiomic analysis of preoperative MRI. Cancers (Basel).

[b0035] Chen C., He Z.C., Shi Y. (2018). Microvascular fractal dimension predicts prognosis and response to chemotherapy in glioblastoma: an automatic image analysis study. Lab. Invest..

[b0040] Choi K.Y., Jung T.Y., Jung S. (2011). Prognosis of oligodendroglial tumor with ring enhancement showing central necrotic portion. J. Neurooncol.

[b0045] D'Amico R.S., Englander Z.K., Canoll P., Bruce J.N. (2017). Extent of resection in glioma-a review of the cutting edge. World Neurosurg..

[b0050] Di Ieva A., Davidson J.M., Russo C. (2024). Computational fractal-based neurosurgery. Adv. Exp. Med. Biol..

[b0055] Drexler R., Lim M., Hervey-Jumper S.L. (2025). Molecular-based decision-making in glioblastoma surgery: when to aim for supramaximal resection. Neuro Oncol..

[b0060] Evleksiz D., Gungor A., Karımzada G. (2025). The mystery of the claustrum, the front wall of the brain: from early anatomic discovery to modern insights. World Neurosurg..

[b0065] Fisher B.J., Naumova E., Leighton C.C. (2002). Ki-67: a prognostic factor for low-grade glioma?. Int. J. Radiat. Oncol. Biol. Phys..

[b0070] Gogia B., Chavali L.S., Lang F.F. (2018). MRI venous architecture of insula. J. Neurol. Sci..

[b0075] Hameed N.U.F., Qiu T., Zhuang D. (2018). Transcortical insular glioma resection: clinical outcome and predictors. J. Neurosurg..

[b0080] Hervey-Jumper S.L., Berger M.S. (2016). Maximizing safe resection of low- and high-grade glioma. J. Neurooncol.

[b0085] Huang Y., Cao Y., Chen H. (2025). Quantifying tumor morphological complexity based on pretreatment MRI fractal analysis for predicting pathologic complete response and survival in breast cancer: a retrospective, multicenter study. Breast Cancer Res..

[b0090] Hui D., Park M., Liu D. (2016). Clinician prediction of survival versus the palliative prognostic score: which approach is more accurate?. Eur. J. Cancer.

[b0095] Kerkhof M., Vecht C.J. (2013). Seizure characteristics and prognostic factors of gliomas. Epilepsia.

[b0100] Lambin P., Rios-Velazquez E., Leijenaar R. (2012). Radiomics: extracting more information from medical images using advanced feature analysis. Eur. J. Cancer.

[b0105] Li Z., Li G., Liu Z. (2020). Transcortical approach for insular gliomas: a series of 253 patients. J. Neurooncol.

[b0110] Li G., Yin C., Zhang C. (2023). Spatial distribution of supratentorial diffuse gliomas: a retrospective study of 990 cases. Front. Oncol..

[b0115] Liu X., Hou Z., He Q. (2025). Quantitative susceptibility mapping study of deep gray matter iron content in glioma patients. Quant. Imaging Med. Surg..

[b0120] Mandelbrot, B.B., The fractal geometry of nature/Revised and enlarged edition. New York. 1983.

[b0125] Marquardt D.W. (1970). Generalized inverses, ridge regression, biased linear estimation, and nonlinear estimation. Technometrics.

[b0130] Michaud K., Duffau H. (2016). Surgery of insular and paralimbic diffuse low-grade gliomas: technical considerations. J. Neurooncol.

[b0135] Morioka S., Kawaguchi R., Yamada Y., Iwai K., Yoshimoto C., Kobayashi H. (2019). Magnetic resonance imaging findings for discriminating clear cell carcinoma and endometrioid carcinoma of the ovary. J. Ovarian. Res..

[b0140] Nachtergaele P., Radwan A., Swinnen S. (2019). The temporoinsular projection system: an anatomical study. J. Neurosurg..

[b0145] Pandith A.A., Zahoor W., Manzoor U. (2023). Evaluation of chromosome 1p/19q deletion by Fluorescence in Situ Hybridization (FISH) as prognostic factors in malignant glioma patients on treatment with alkylating chemotherapy. Cancer Genet..

[b0150] Patel S.H., Poisson L.M., Brat D.J. (2017). T2-FLAIR mismatch, an imaging biomarker for IDH and 1p/19q status in lower-grade gliomas: a TCGA/TCIA project. Clin. Cancer Res..

[b0155] Pérez-Beteta J., Molina-García D., Ortiz-Alhambra J.A. (2018). Tumor surface regularity at MR imaging predicts survival and response to surgery in patients with glioblastoma. Radiology.

[b0160] Philip N.T., Bolem S., Sudhir B.J., Patnaik B.S.V. (2022). Hemodynamics and bio-mechanics of morphologically distinct saccular intracranial aneurysms at bifurcations: idealised vs patient-specific geometries. Comput. Methods Programs Biomed..

[b0165] Przybylowski C.J., Hervey-Jumper S.L., Sanai N. (2021). Surgical strategy for insular glioma. J. Neurooncol.

[b0170] Reuss D.E., Sahm F., Schrimpf D. (2015). ATRX and IDH1-R132H immunohistochemistry with subsequent copy number analysis and IDH sequencing as a basis for an “integrated” diagnostic approach for adult astrocytoma, oligodendroglioma and glioblastoma. Acta Neuropathol..

[b0175] Saad M., Lee I.H., Choi T.S. (2019). Are shape morphologies associated with survival? a potential shape-based biomarker predicting survival in lung cancer. J. Cancer Res. Clin. Oncol..

[b0180] Sanai N., Polley M.Y., Berger M.S. (2010). Insular glioma resection: assessment of patient morbidity, survival, and tumor progression. J. Neurosurg..

[b0185] Sánchez J., Martín-Landrove M. (2022). Morphological and fractal properties of brain tumors. Front. Physiol..

[b0190] Shahzadi I., Seidlitz A., Beuthien-Baumann B. (2024). Radiomics for residual tumour detection and prognosis in newly diagnosed glioblastoma based on postoperative [11C] methionine PET and T1c-w MRI. Sci. Rep..

[b0195] Shi D.D., Savani M.R., Levitt M.M. (2022). De novo pyrimidine synthesis is a targetable vulnerability in IDH mutant glioma. Cancer Cell.

[b0200] Smitha K.A., Gupta A.K., Jayasree R.S. (2015). Fractal analysis: fractal dimension and lacunarity from MR images for differentiating the grades of glioma. Phys. Med. Biol..

[b0205] Su C., Jiang J., Zhang S. (2019). Radiomics based on multicontrast MRI can precisely differentiate among glioma subtypes and predict tumour-proliferative behaviour. Eur. Radiol..

[b0210] Takami H., Yoshida A., Fukushima S. (2015). Revisiting TP53 mutations and immunohistochemistry–a comparative study in 157 diffuse gliomas. Brain Pathol..

[b0215] Türe U., Yaşargil M.G., Al-Mefty O., Yaşargil D.C. (2000). Arteries of the insula. J. Neurosurg..

[b0220] van Garderen K.A., Vallentgoed W.R., Lavrova A. (2023). Longitudinal characteristics of T2-FLAIR mismatch in IDH-mutant astrocytomas: relation to grade, histopathology, and overall survival in the GLASS-NL cohort. Neurooncol. Adv..

[b0225] Varnavas G.G., Grand W. (1999). The insular cortex: morphological and vascular anatomic characteristics. Neurosurgery.

[b0230] Villanueva-Meyer J.E., Wood M.D., Choi B.S. (2018). MRI features and IDH mutational status of grade II diffuse gliomas: impact on diagnosis and prognosis. AJR Am. J. Roentgenol..

[b0235] Weller M., van den Bent M., Preusser M. (2021). EANO guidelines on the diagnosis and treatment of diffuse gliomas of adulthood. Nat. Rev. Clin. Oncol..

[b0240] Wiestler B., Capper D., Holland-Letz T. (2013). ATRX loss refines the classification of anaplastic gliomas and identifies a subgroup of IDH mutant astrocytic tumors with better prognosis. Acta Neuropathol..

[b0245] Xie J., Li C., Liu F. (2011). Resection of insular glioma via the trans-Sylvian approach [in Chinese]. Zhonghua Shenjing Waike Zazhi (Chin. J. Neurosurg.).

[b0250] Xie H., Tubbs R., Yang B. (2015). Detection of MGMT promoter methylation in glioblastoma using pyrosequencing. Int. J. Clin. Exp. Path..

[b0255] Xue J., Liu H., Jiang L., Yin Q., Chen L., Wang M. (2025). Limitations of nomogram models in predicting survival outcomes for glioma patients. Front. Immunol..

[b0260] Yang G., Jones T.L., Howe F.A., Barrick T.R. (2016). Morphometric model for discrimination between glioblastoma multiforme and solitary metastasis using three-dimensional shape analysis. Magn. Reson. Med..

[b0265] Yang Z.C., Xue B.W., Song X.Y. (2024). Connectomic insights into the impact of 1p/19q co-deletion in dominant hemisphere insular glioma patients. Front. Neurosci..

