# Supplementary figures and images for: The specific linear or curved boundaries between WHO grade II–III insular gliomas and the basal ganglia indicate distinct biological features, survival outcomes, and surgical strategies: evidence from 330 cases
Source: Neuroimage Clin. 2026 Apr 25;50:103995. doi: 10.1016/j.nicl.2026.103995 (PMC13141764; doi:10.1016/j.nicl.2026.103995)

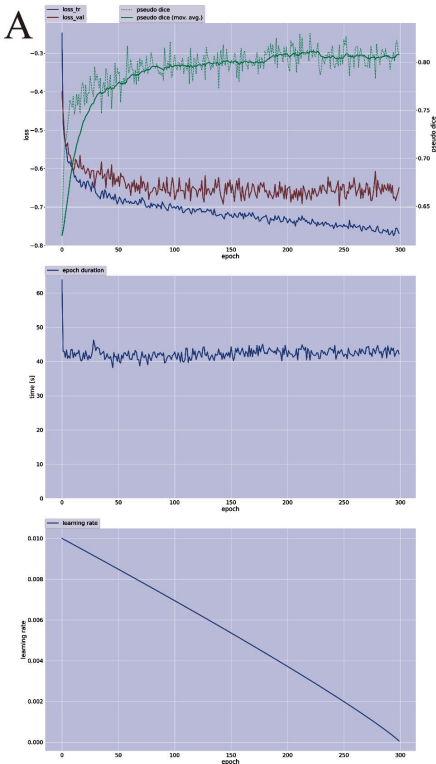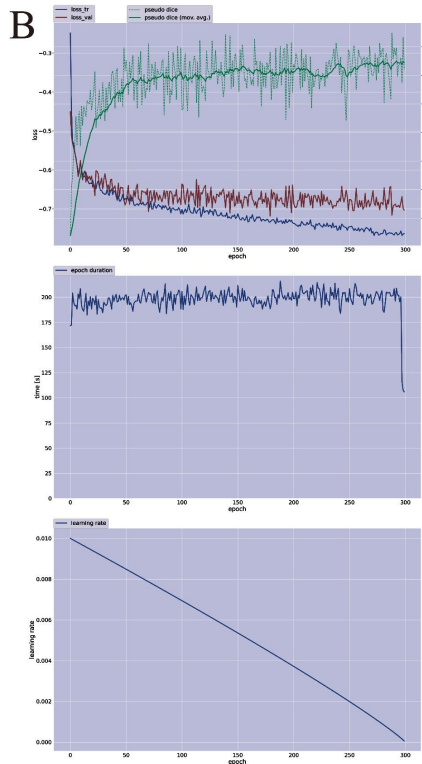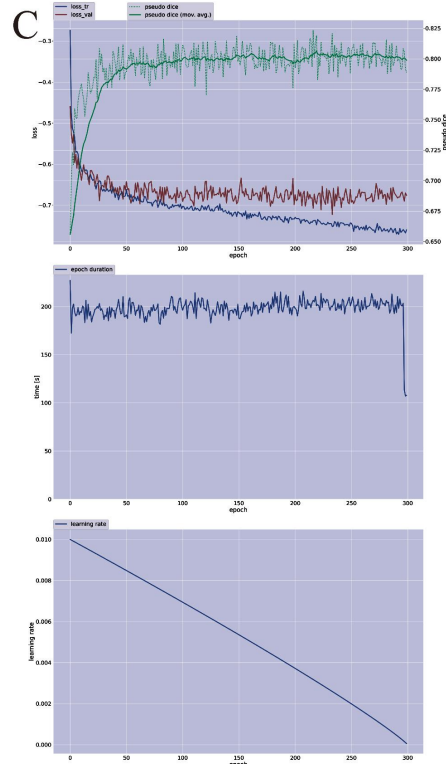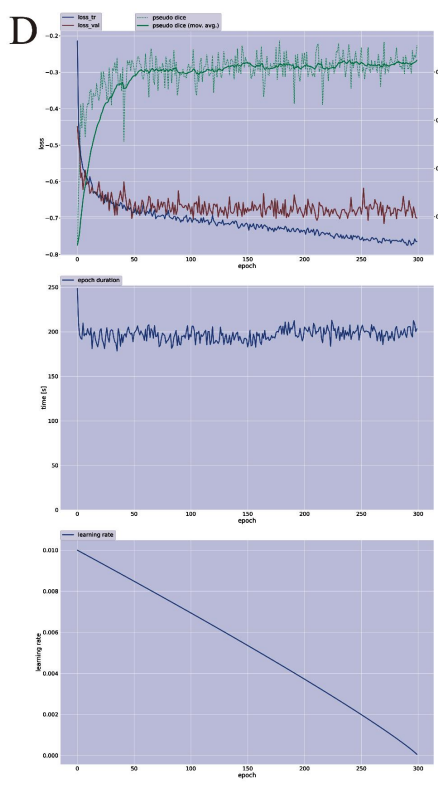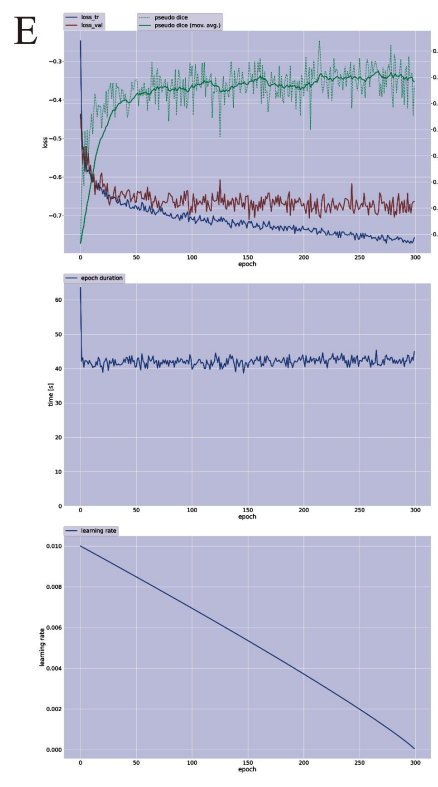

Supplement: Supplementary Fig. S1 — Learning curve of the model. The U-Net_v2 deep learning model was trained using the Medical Segmentation Decathlon (http://medicaldecathlon.com/). All data from Task01_BrainTumour were used for training with 5-fold cross-validation and 200 epochs. The best model was utilized to conduct tumor segmentation. The hardware environment was based on Ubuntu 22.04 (Canonical Ltd., UK) operating system, equipped with a 14-core CPU, 32GB RAM, and an NVIDIA RTX 4090 GPU (24GB VRAM). The software environment was built on Python (version 3.12, Python Software Foundation, USA), and PyTorch (version 2.3.0, PyTorch Foundation, USA) with CUDA (version 12.1, NVIDIA, USA) support. The nnUNet_v2 framework was installed within an Anaconda virtual environment. [file mmc1.pdf]

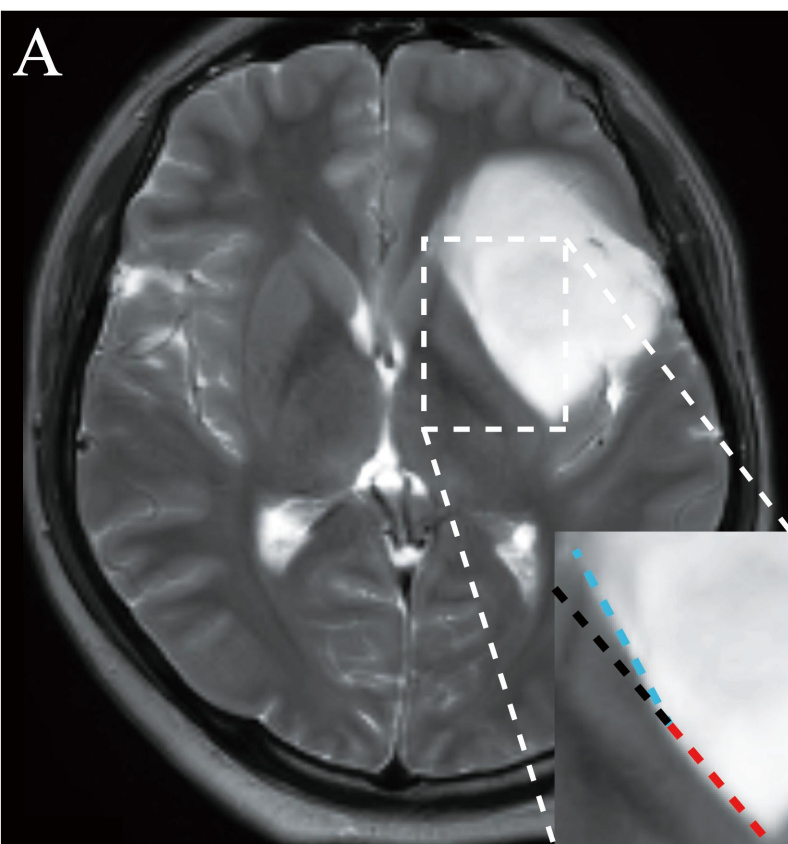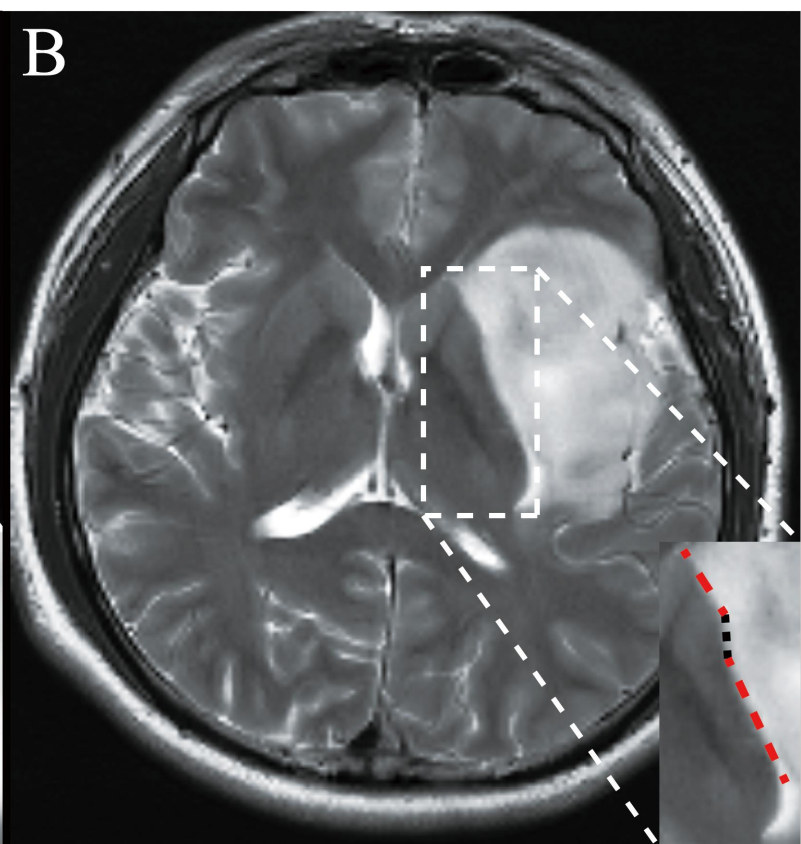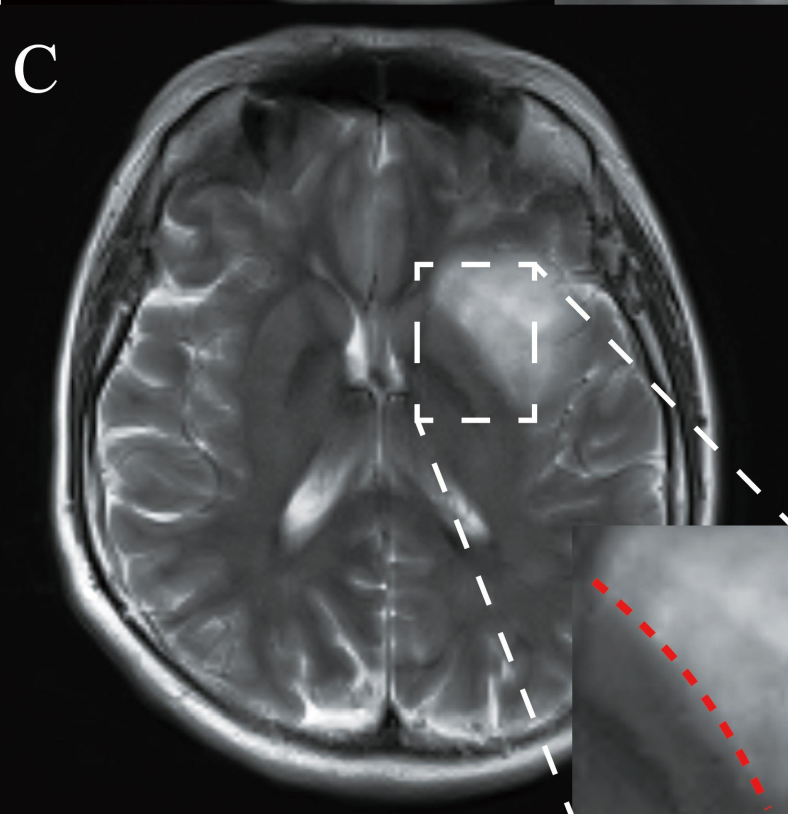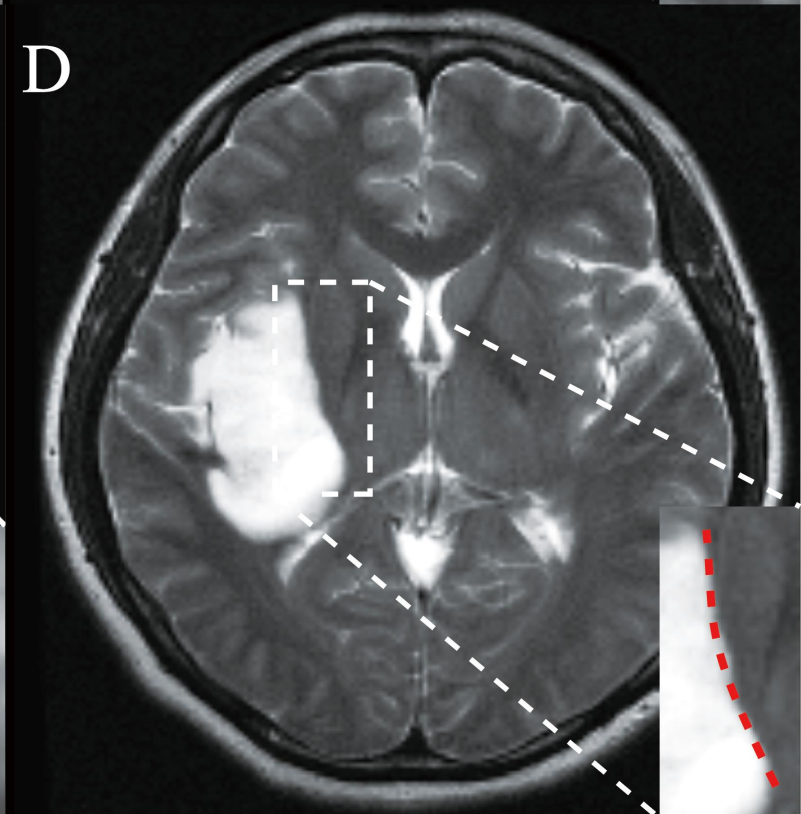

Supplement: Supplementary Fig. S2 — Special cases in the fractal dimension measured process. A: The insular glioma did not advance uniformly inward, possibly due to tumor heterogeneity; B: the part of insular glioma did advanced inward, which may be caused by tumor heterogeneity; C: The insular glioma may occur in the anterior insular lobe; D: The insular glioma may locate in the posterior insular lobe. [file mmc2.pdf]

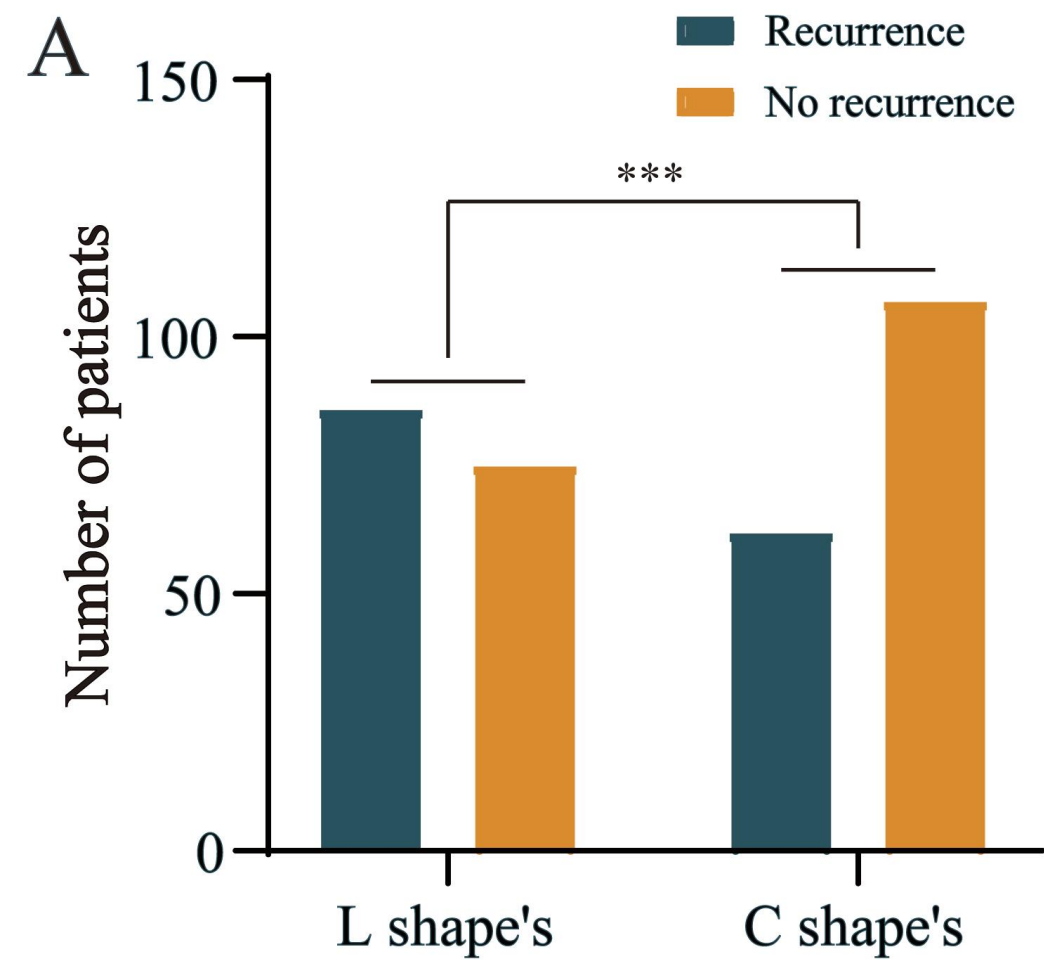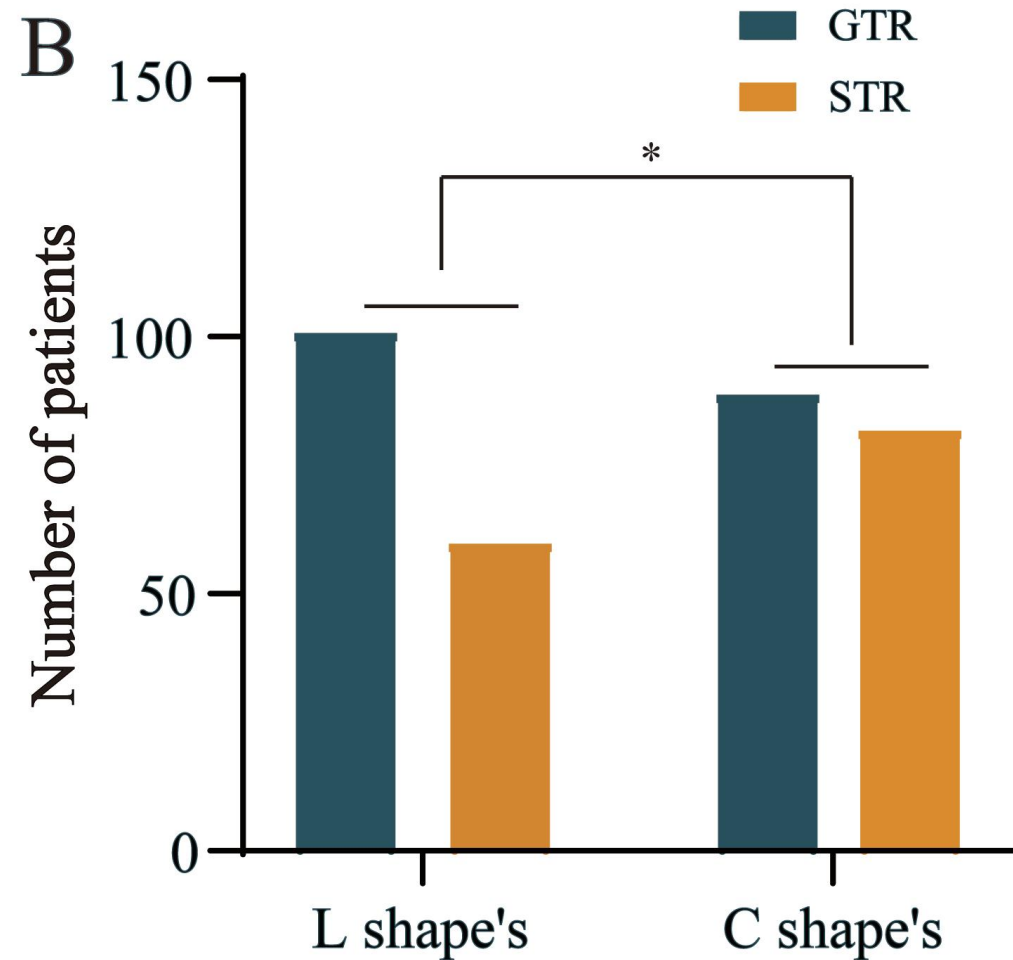

Supplement: Supplementary Fig. S3 — Analysis of recurrence and extent of resection. A: Recurrence risk between the L and C subgroups; B: Proportion of GTR and STR in L and C subgroups. Abbreviations: L: linear; C: curved; GTR: gross total resection; STR: subtotal resection; *: p < 0.05; **: p < 0.01; ***: p < .001. [file mmc3.pdf]

**A**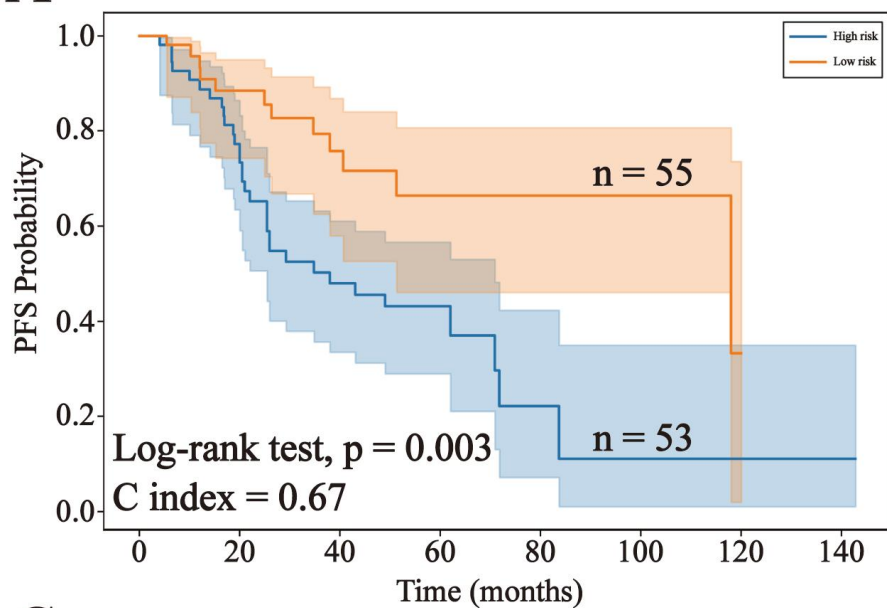**B**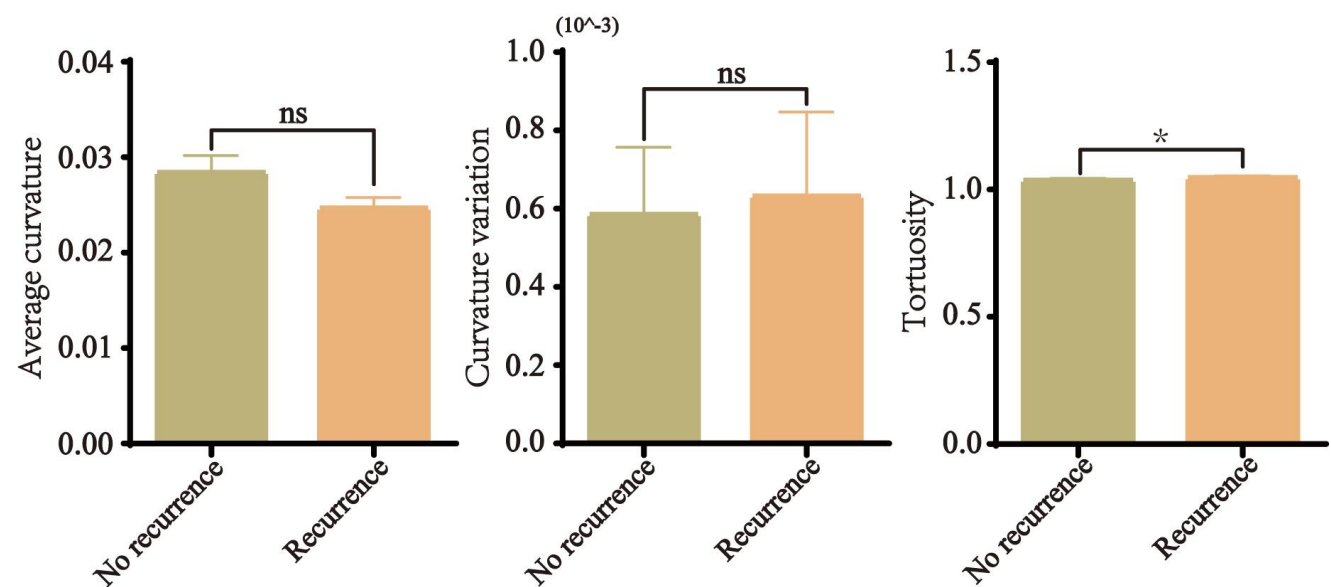**C**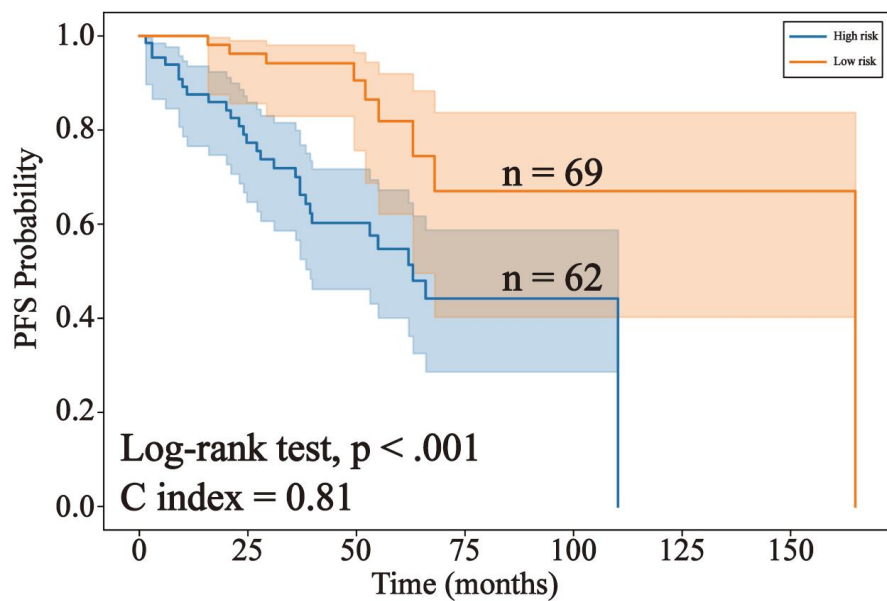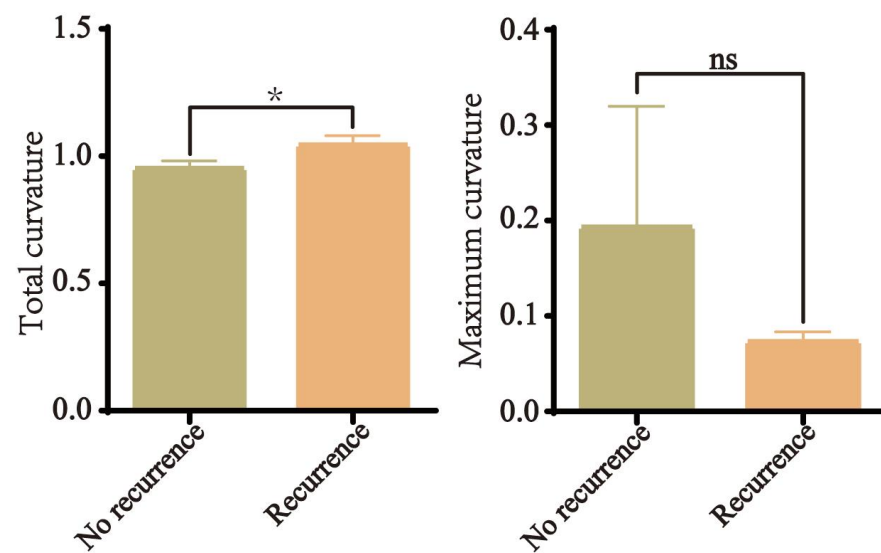

Supplement: Supplementary Fig. S4 — Disease stratification based on the statistical regression model. A: Kaplan–Meier analysis of PFS between the high- and low- risk cohorts within L subgroup; B: Analysis of related parameters about curved shape; C: Kaplan–Meier analysis of PFS between the high- and low- risk cohorts within C subgroup. Abbreviations: PFS: progression-free survival; L: linear; C: curved; p: p value; n: number. [file mmc4.pdf]

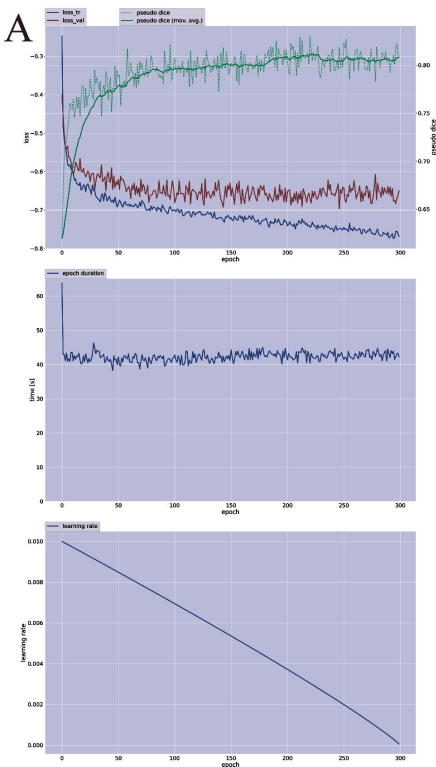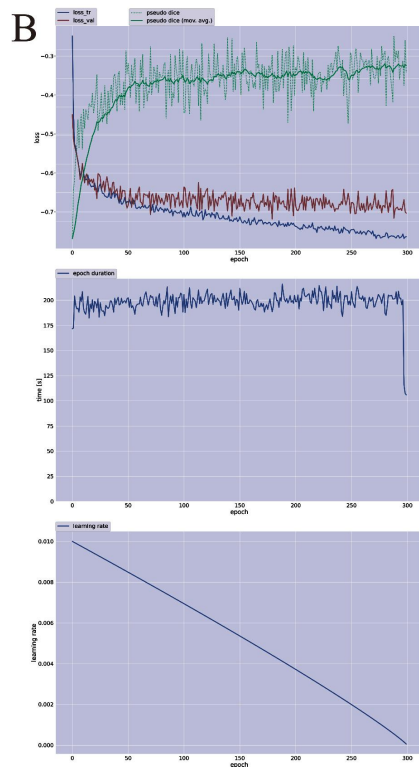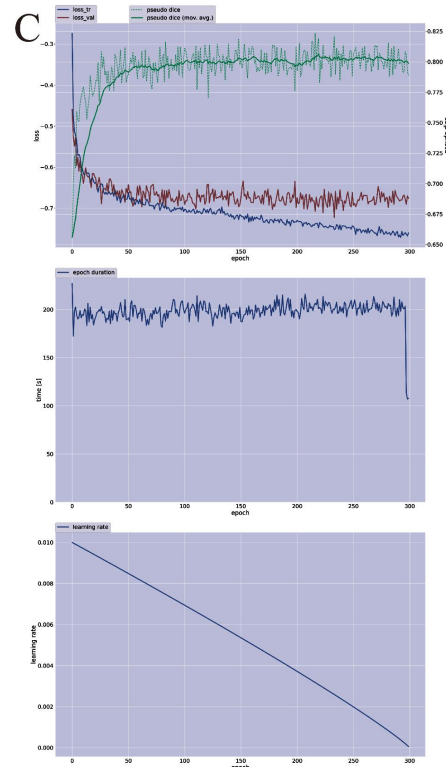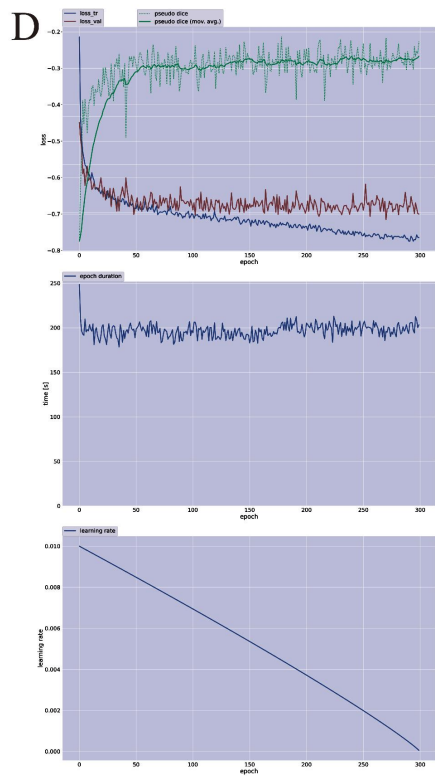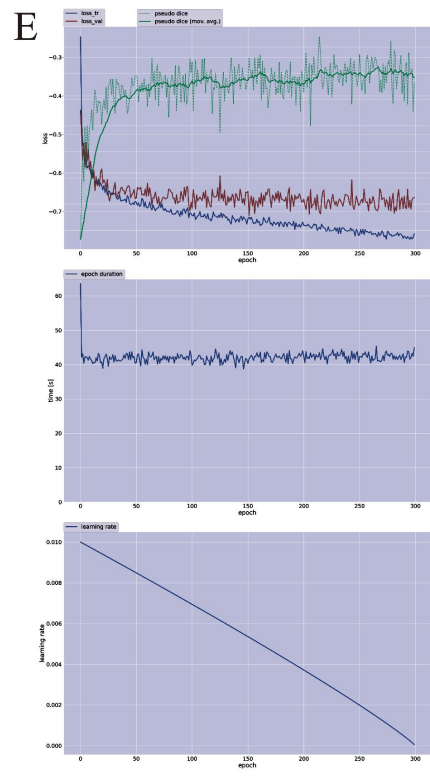

Supplement: Supplementary Data 5 [file mmc5.pdf]

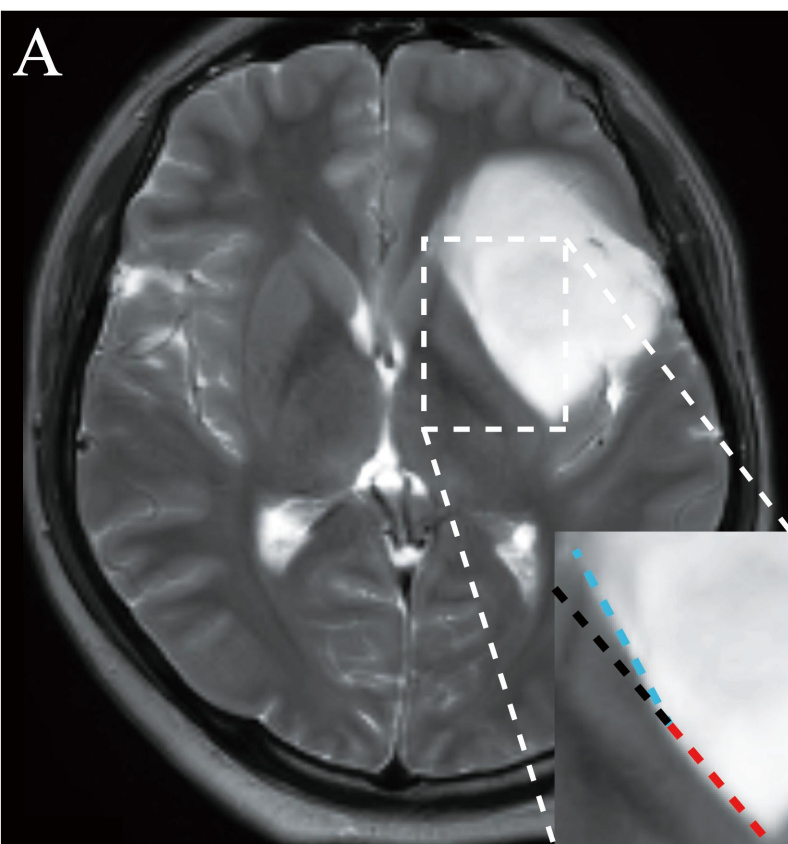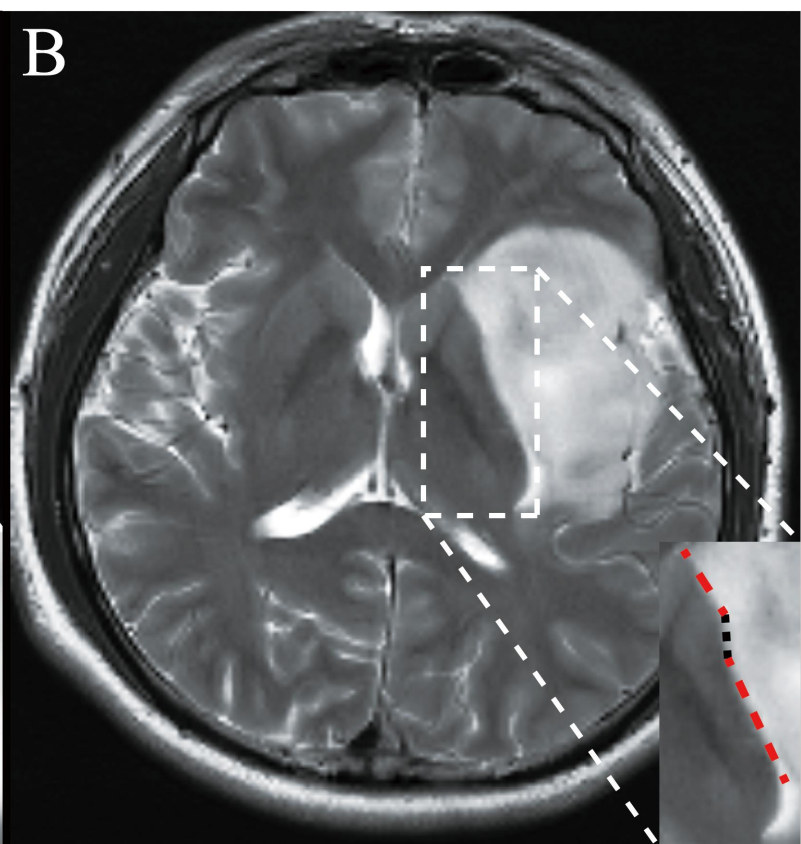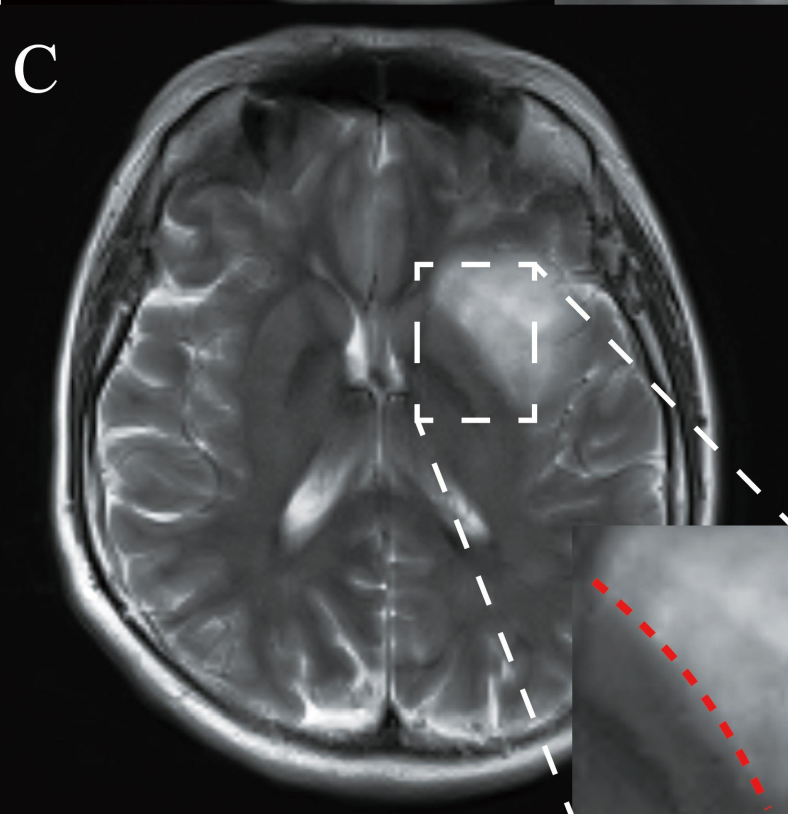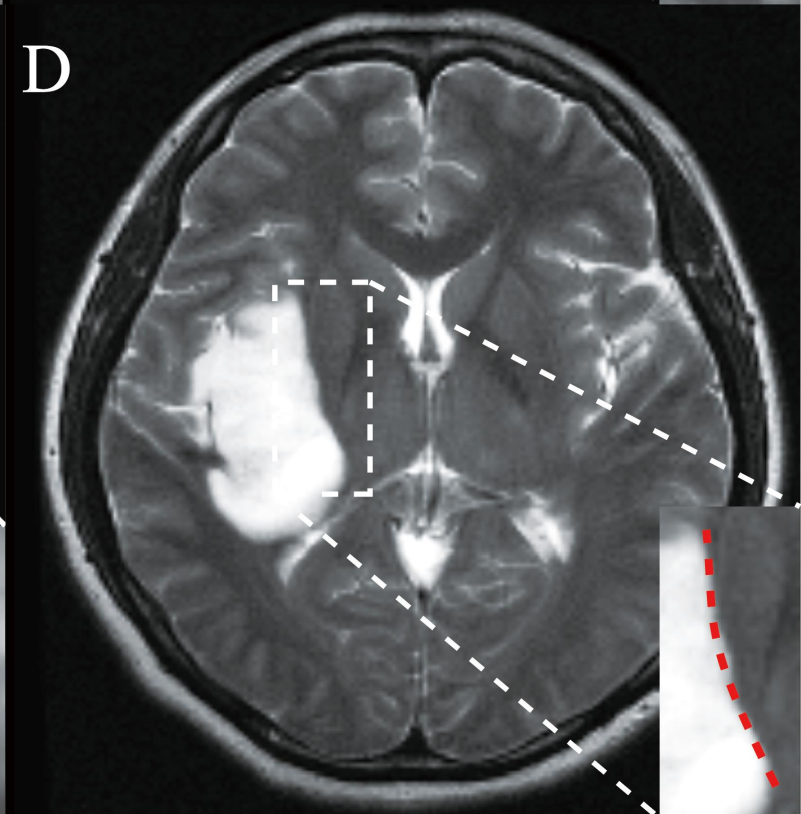

Supplement: Supplementary Data 6 [file mmc6.pdf]

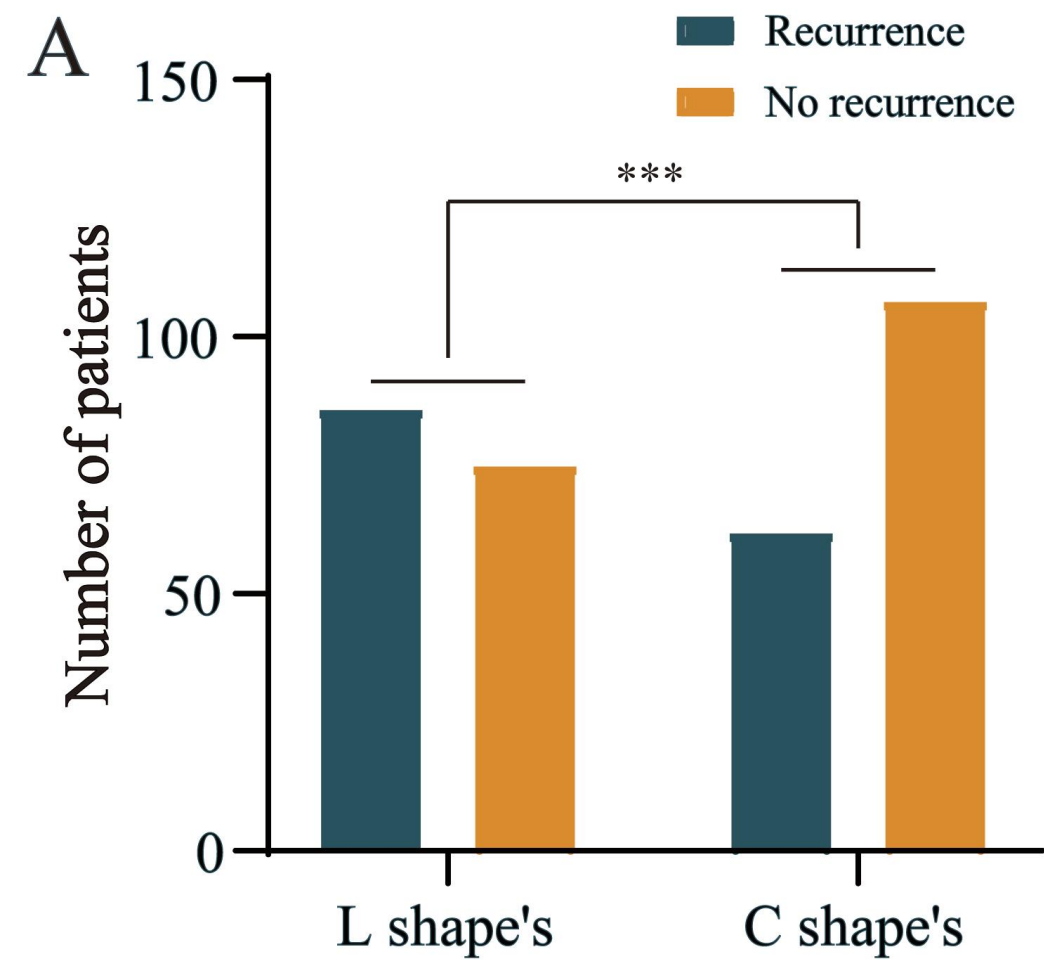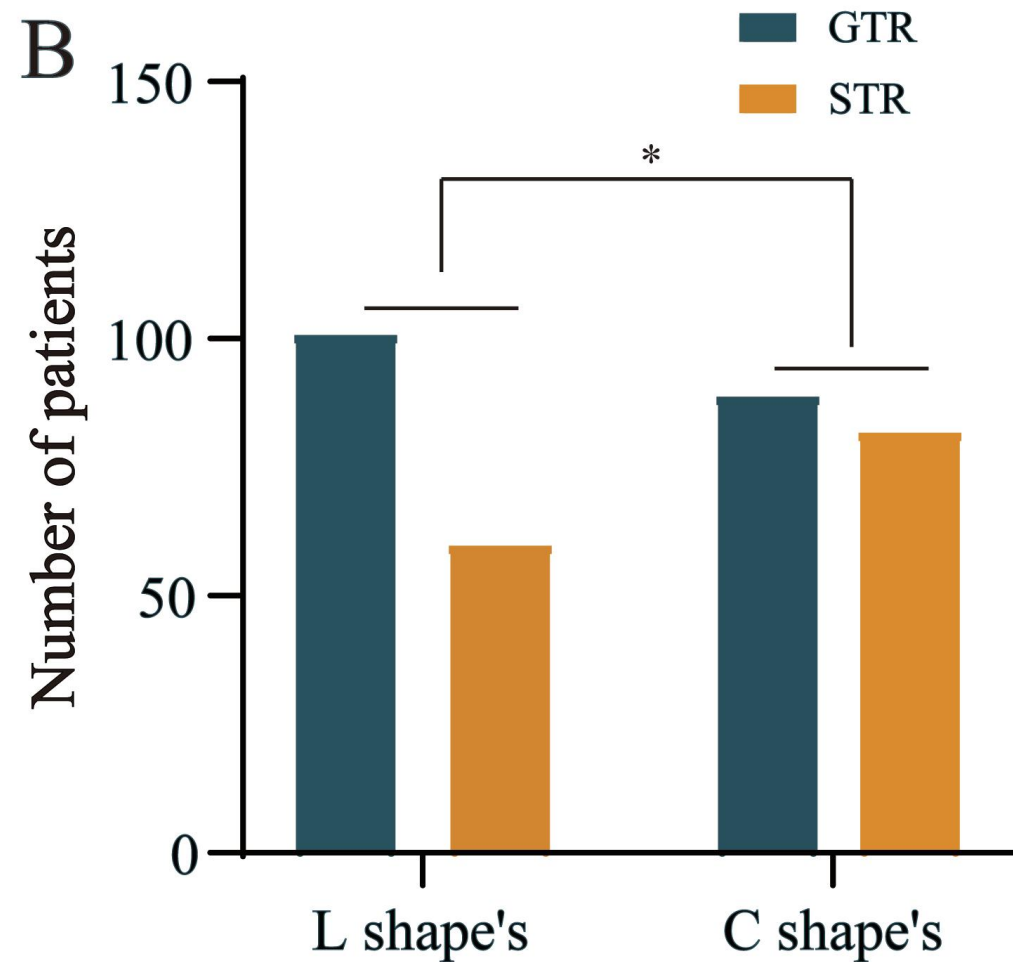

Supplement: Supplementary Data 7 [file mmc7.pdf]

**A**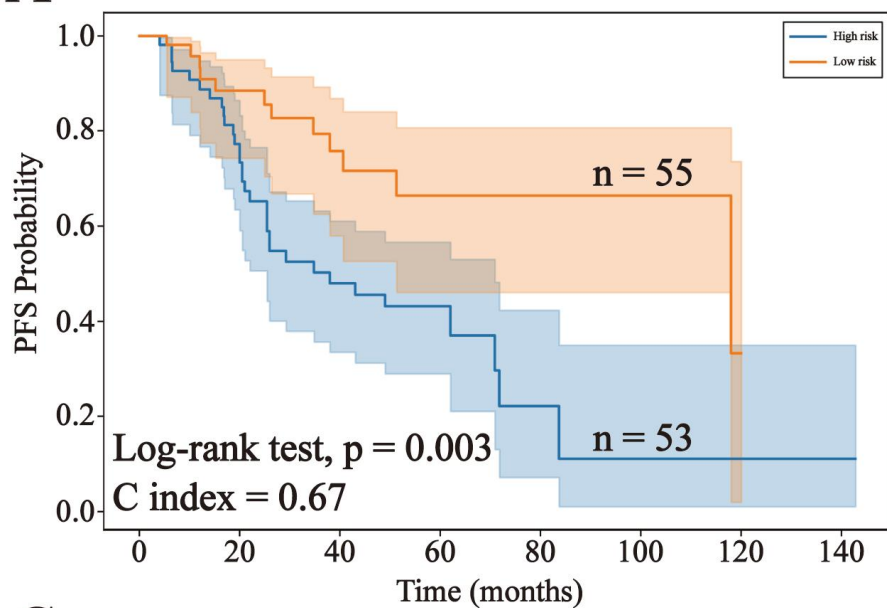**B**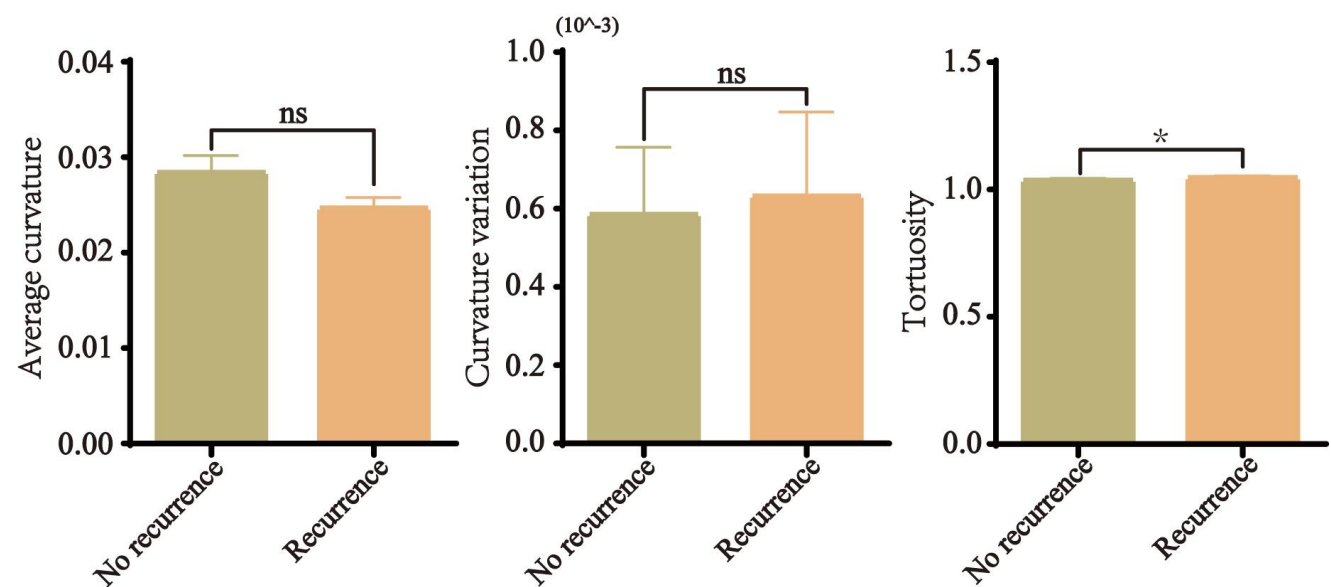**C**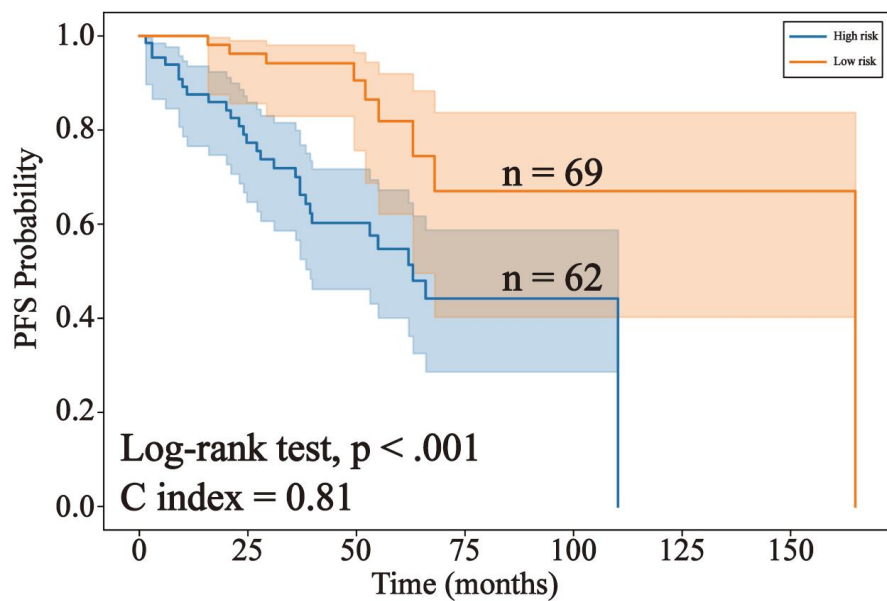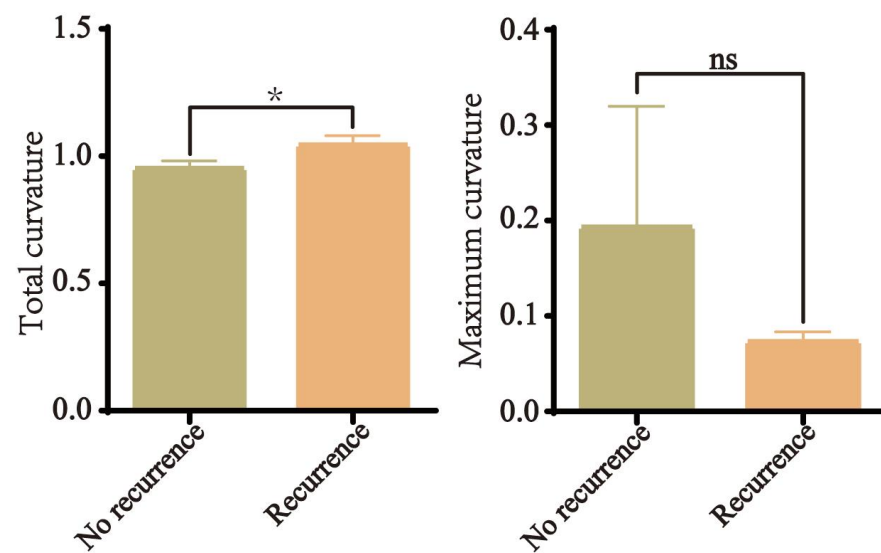

Supplement: Supplementary Data 8 [file mmc8.pdf]
