# Supplementary material for: The specific linear or curved boundaries between WHO grade II–III insular gliomas and the basal ganglia indicate distinct biological features, survival outcomes, and surgical strategies: evidence from 330 cases
Source: Neuroimage Clin. 2026 Apr 25;50:103995. doi: 10.1016/j.nicl.2026.103995 (PMC13141764; doi:10.1016/j.nicl.2026.103995)
Supplement: Supplementary Data 9 [file mmc9.docx]

**Supplement Table S1. MR Imaging Parameters**

| **Sequence type** | **TR (ms)** | **TE (ms)** | **TI (ms)** | **Flip angle (°)** | **Matrix size** | **FOV (mm)** | **Slice thickness (mm)** | **Notes** |
| --- | --- | --- | --- | --- | --- | --- | --- | --- |
| 3D T1-weighted (MPRAGE) | 1540 | 2.4 | 900 | 8 | 256 × 232 | 217 × 240 | 1 | Pre-contrast |
| T2-weighted (TSE) | 4500 | 105 | N/A | 150 | 448 × 283 | 185 × 220 | 5 | N/A |
| FLAIR (TSE) | 6000 | 81 | 2028.3 | 90 | 320 × 196 | 192 × 220 | 5 | Fluid-attenuated sequence |
| Contrast-enhanced 3D T1-weighted | 1540 | 2.4 | N/A | 8 | 256 × 232 | N/A | 1 | Gadobenate dimeglumine (0.1 mmol/kg, 4.0 mL/s + 30 mL saline flush) |
