# Supplementary material for: The specific linear or curved boundaries between WHO grade II–III insular gliomas and the basal ganglia indicate distinct biological features, survival outcomes, and surgical strategies: evidence from 330 cases
Source: Neuroimage Clin. 2026 Apr 25;50:103995. doi: 10.1016/j.nicl.2026.103995 (PMC13141764; doi:10.1016/j.nicl.2026.103995)
Supplement: Supplementary Data 10 [file mmc10.docx]

**Supplement Table S2. The matrix of Spearman's rank correlation coefficient analysis**

| **Variate** | **Sex** | **Age** | **Side** | **Tumor volume** | **History**  **of**  **epilepsy** | **Histological**  **type** | **WHO**  **grade** | | **IDH1**  **status** | **1p/19q**  **status** | **IDH1^+^,**  **1p/19q**  **status** | **MGMT**  **status** | **ATRX**  **status** | **P53**  **status** | **Ki-67**  **index** |
| --- | --- | --- | --- | --- | --- | --- | --- | --- | --- | --- | --- | --- | --- | --- | --- |
| **Sex** | 1.000 | 0.022 | -0.030 | -0.039 | 0.060 | 0.070 | 0.063 | -0.107 | | 0.100 | 0.163 | 0.045 | -0.079 | -0.001 | -0.075 |
| **Age** | 0.022 | 1.000 | 0.046 | -0.039 | -0.017 | 0.156 | 0.035 | -0.087 | | 0.047 | 0.139 | 0.008 | -0.216 | 0.007 | 0.151 |
| **Side** | -0.030 | 0.046 | 1.000 | -0.010 | -0.058 | 0.225 | -0.150 | 0.030 | | -0.129 | -0.054 | 0.043 | -0.088 | 0.046 | 0.284 |
| **Tumor volume** | -0.039 | -0.039 | -0.010 | 1.000 | 0.029 | -0.119 | -0.054 | -0.057 | | -0.007 | 0.048 | -0.060 | 0.017 | 0.016 | 0.083 |
| **History of epilepsy** | 0.060 | -0.017 | -0.058 | 0.029 | 1.000 | -0.015 | -0.075 | -0.123 | | 0.023 | 0.056 | 0.041 | -0.034 | 0.018 | -0.004 |
| **Histological type** | 0.070 | 0.156 | 0.225 | -0.119 | -0.015 | 1.000 | -0.121 | 0.158 | | 0.167 | 0.318 | 0.169 | -0.175 | -0.047 | 0.351 |
| **WHO grade** | 0.063 | 0.035 | -0.150 | -0.054 | -0.075 | -0.121 | 1.000 | 0.056 | | 0.119 | -0.081 | 0.016 | 0.058 | 0.097 | -0.132 |
| **IDH1 status** | -0.107 | -0.087 | 0.030 | -0.057 | -0.123 | 0.158 | 0.056 | 1.000 | | 0.015 | -0.156 | 0.202 | 0.378 | -0.020 | 0.212 |
| **1p/19q status** | 0.100 | 0.047 | -0.129 | -0.007 | 0.023 | 0.167 | 0.119 | 0.015 | | 1.000 | 0.528 | 0.048 | -0.044 | -0.039 | -0.125 |
| **IDH1^+^, 1p/19q status** | 0.163 | 0.139 | -0.054 | 0.048 | 0.056 | 0.318 | -0.081 | -0.156 | | 0.528 | 1.000 | 0.034 | -0.239 | -0.025 | 0.030 |
| **MGMT status** | 0.045 | 0.008 | 0.043 | -0.060 | 0.041 | 0.169 | 0.016 | 0.202 | | 0.048 | 0.034 | 1.000 | 0.115 | 0.008 | 0.113 |
| **ATRX status** | -0.079 | -0.216 | -0.088 | 0.017 | -0.034 | -0.175 | 0.058 | 0.378 | | -0.044 | -0.239 | 0.115 | 1.000 | 0.043 | 0.071 |
| **P53 status** | -0.001 | 0.007 | 0.046 | 0.016 | 0.018 | -0.047 | 0.097 | -0.020 | | -0.039 | -0.025 | 0.008 | 0.043 | 1.000 | 0.109 |
| **Ki-67 index** | -0.075 | 0.151 | 0.284 | 0.083 | -0.004 | 0.351 | -0.132 | 0.212 | | -0.125 | 0.030 | 0.113 | 0.071 | 0.109 | 1.000 |

**Abbreviations: The best cut-off value of age, tumor volume was 38 years and 20.17 cm^3^, respectively. WHO: World Health Organization; IDH1: Isocitrate dehydrogenase 1; 1p/19q co-delete: co-deletion of chromosomal arms 1p and 19q; MGMT: O_6_-methylguanine-DNA methyltransferase; ATRX: Alpha thalassemia/mental retardation syndrome X-linked; TP53: Tumor protein p53; Ki-67: Ki-67 labeling index; IDH1+: IDH1 mutation**
