# Supplementary material for: The specific linear or curved boundaries between WHO grade II–III insular gliomas and the basal ganglia indicate distinct biological features, survival outcomes, and surgical strategies: evidence from 330 cases
Source: Neuroimage Clin. 2026 Apr 25;50:103995. doi: 10.1016/j.nicl.2026.103995 (PMC13141764; doi:10.1016/j.nicl.2026.103995)
Supplement: Supplementary Data 11 [file mmc11.docx]

**Supplement Table S3. The matrix of Spearman's rank correlation analysis’ p value**

| **Variate** | **Sex** | **Age** | **Side** | **Tumor volume** | **History**  **of**  **epilepsy** | **Histological**  **type** | **WHO**  **grade** | | **IDH1**  **status** | **1p/19q**  **status** | **IDH1+,**  **1p/19q**  **status** | **MGMT**  **status** | **ATRX**  **status** | **P53**  **status** | **Ki-67**  **index** |
| --- | --- | --- | --- | --- | --- | --- | --- | --- | --- | --- | --- | --- | --- | --- | --- |
| **Sex** | 0.000 | 0.692 | 0.591 | 0.478 | 0.274 | 0.207 | 0.252 | 0.051 | | 0.069 | 0.003 | 0.412 | 0.151 | 0.991 | 0.172 |
| **Age** | 0.692 | 0.000 | 0.402 | 0.477 | 0.755 | 0.005 | 0.524 | 0.115 | | 0.390 | 0.011 | 0.889 | 0.000 | 0.892 | 0.006 |
| **Side** | 0.591 | 0.402 | 0.000 | 0.857 | 0.293 | 0.000 | 0.006 | 0.589 | | 0.019 | 0.331 | 0.441 | 0.113 | 0.401 | 0.000 |
| **Tumor volume** | 0.478 | 0.477 | 0.857 | 0.000 | 0.595 | 0.030 | 0.331 | 0.301 | | 0.897 | 0.386 | 0.276 | 0.761 | 0.772 | 0.134 |
| **History of epilepsy** | 0.274 | 0.755 | 0.293 | 0.595 | 0.000 | 0.784 | 0.171 | 0.026 | | 0.673 | 0.312 | 0.453 | 0.539 | 0.749 | 0.941 |
| **Histological type** | 0.207 | 0.005 | 0.000 | 0.030 | 0.784 | 0.000 | 0.028 | 0.004 | | 0.002 | 0.000 | 0.002 | 0.001 | 0.391 | 0.000 |
| **WHO grade** | 0.252 | 0.524 | 0.006 | 0.331 | 0.171 | 0.028 | 0.000 | 0.310 | | 0.031 | 0.144 | 0.772 | 0.294 | 0.079 | 0.017 |
| **IDH1 status** | 0.051 | 0.115 | 0.589 | 0.301 | 0.026 | 0.004 | 0.310 | 0.000 | | 0.787 | 0.004 | 0.000 | 0.000 | 0.722 | 0.000 |
| **1p/19q status** | 0.069 | 0.390 | 0.019 | 0.897 | 0.673 | 0.002 | 0.031 | 0.787 | | 0.000 | 0.000 | 0.383 | 0.422 | 0.482 | 0.023 |
| **IDH1^+^, 1p/19q status** | 0.003 | 0.011 | 0.331 | 0.386 | 0.312 | 0.000 | 0.144 | 0.004 | | 0.000 | 0.000 | 0.542 | 0.000 | 0.646 | 0.585 |
| **MGMT status** | 0.412 | 0.889 | 0.441 | 0.276 | 0.453 | 0.002 | 0.772 | 0.000 | | 0.383 | 0.542 | 0.000 | 0.036 | 0.889 | 0.041 |
| **ATRX status** | 0.151 | 0.000 | 0.113 | 0.761 | 0.539 | 0.001 | 0.294 | 0.000 | | 0.422 | 0.000 | 0.036 | 0.000 | 0.433 | 0.200 |
| **P53 status** | 0.991 | 0.892 | 0.401 | 0.772 | 0.749 | 0.391 | 0.079 | 0.722 | | 0.482 | 0.646 | 0.889 | 0.433 | 0.000 | 0.047 |
| **Ki-67 index** | 0.172 | 0.006 | 0.000 | 0.134 | 0.941 | 0.000 | 0.017 | 0.000 | | 0.023 | 0.585 | 0.041 | 0.200 | 0.047 | 0.000 |

**Abbreviations: The best cut-off value of age, tumor volume was 38 years and 20.17 cm3, respectively. WHO: World Health Organization; IDH1: Isocitrate dehydrogenase 1; 1p/19q: chromosomal arms 1p and 19q; MGMT: O6-methylguanine-DNA methyltransferase; ATRX: Alpha thalassemia/mental retardation syndrome X-linked; TP53: Tumor protein p53; Ki-67: Ki-67 labeling index; IDH1+: IDH1 mutation**
