# Supplementary material for: The specific linear or curved boundaries between WHO grade II–III insular gliomas and the basal ganglia indicate distinct biological features, survival outcomes, and surgical strategies: evidence from 330 cases
Source: Neuroimage Clin. 2026 Apr 25;50:103995. doi: 10.1016/j.nicl.2026.103995 (PMC13141764; doi:10.1016/j.nicl.2026.103995)
Supplement: Supplementary Data 13 [file mmc13.docx]

**Supplement Table S5. The result of multivariate COX regression analysis**

| **Variate** | **HR** | **95% CI** | ***p*** |
| --- | --- | --- | --- |
| Sex | 1.02 | 0.94-1.11 | 0.57 |
| Age | 1.00 | 1.00-1.01 | 0.12 |
| Side | 1.61 | 1.46-1.77 | 0.00 |
| Tumor volume | 0.91 | 0.81-1.03 | 0.13 |
| History of epilepsy | 1.01 | 0.93-1.09 | 0.87 |
| Histological classification | 1.26 | 1.13-1.40 | 0.00 |
| WHO grade | 0.89 | 0.82-0.97 | 0.01 |
| IDH1 status | 1.10 | 1.01-1.21 | 0.03 |
| 1p/19q status | 0.95 | 0.86-1.05 | 0.30 |
| IDH1^+^, 1p/19q status | 1.08 | 0.96-1.22 | 0.19 |
| MGMT status | 1.02 | 0.93-1.11 | 0.73 |
| ATRX status | 0.96 | 0.88-1.05 | 0.36 |
| P53 status | 0.99 | 0.91-1.08 | 0.89 |
| Ki-67 index | 1.02 | 1.01-1.02 | 0.00 |

**Abbreviations: HR: Hazard Ratio; WHO: World Health Organization; IDH1: Isocitrate dehydrogenase 1; 1p/19q: chromosomal arms 1p and 19q; MGMT: O6-methylguanine-DNA methyltransferase; ATRX: Alpha thalassemia/mental retardation syndrome X-linked; TP53: Tumor protein p53; Ki-67: Ki-67 labeling index; IDH1^+^: IDH1 mutation; *p*: p value**
