# Supplementary material for: The specific linear or curved boundaries between WHO grade II–III insular gliomas and the basal ganglia indicate distinct biological features, survival outcomes, and surgical strategies: evidence from 330 cases
Source: Neuroimage Clin. 2026 Apr 25;50:103995. doi: 10.1016/j.nicl.2026.103995 (PMC13141764; doi:10.1016/j.nicl.2026.103995)
Supplement: Supplementary Data 18 [file mmc18.docx]

**Supplement Table S10. The matrix of Spearman's rank correlation coefficient analysis in C subgoup**

| **Variates** | **TC** | **Tortuosity** | **Sex** | **Age** | **Side** | **WHO**  **grade** | **IDH1**  **status** | **ATRX**  **status** | **P53**  **status** | **Histological**  **type** | **IDH1^+^,**  **1p/19q**  **status** | **1p/19q**  **status** | **MGMT**  **status** | **Ki-67**  **index** | **TV** | **History of epilepsy** |
| --- | --- | --- | --- | --- | --- | --- | --- | --- | --- | --- | --- | --- | --- | --- | --- | --- |
| **TC** | 1.00 | 0.57 | -0.04 | 0.07 | -0.09 | 0.17 | -0.04 | 0.04 | 0.04 | -0.06 | -0.04 | -0.01 | -0.01 | -0.05 | 0.05 | 0.10 |
| **Tortuosity** | 0.57 | 1.00 | 0.05 | 0.08 | 0.07 | 0.07 | -0.12 | -0.05 | 0.01 | -0.03 | -0.08 | -0.05 | -0.10 | -0.08 | 0.03 | 0.24 |
| **Sex** | -0.04 | 0.05 | 1.00 | -0.02 | -0.03 | -0.02 | -0.18 | -0.20 | -0.12 | 0.09 | 0.22 | 0.22 | 0.02 | -0.09 | 0.02 | 0.07 |
| **Age** | 0.07 | 0.08 | -0.02 | 1.00 | -0.12 | 0.18 | -0.08 | -0.14 | -0.03 | 0.01 | 0.09 | 0.11 | -0.05 | 0.05 | -0.03 | 0.04 |
| **Side** | -0.09 | 0.07 | -0.03 | -0.12 | 1.00 | -0.12 | -0.08 | -0.05 | 0.06 | -0.01 | -0.16 | -0.16 | 0.00 | -0.06 | 0.01 | -0.07 |
| **WHO grade** | 0.17 | 0.07 | -0.02 | 0.18 | -0.12 | 1.00 | -0.18 | -0.10 | 0.07 | -0.23 | 0.01 | 0.01 | -0.17 | 0.33 | 0.18 | 0.02 |
| **IDH1 status** | -0.04 | -0.12 | -0.18 | -0.08 | -0.08 | -0.18 | 1.00 | 0.33 | 0.15 | 0.25 | -0.06 | -0.06 | 0.26 | 0.18 | -0.30 | -0.23 |
| **ATRX status** | 0.04 | -0.05 | -0.20 | -0.14 | -0.05 | -0.10 | 0.33 | 1.00 | 0.42 | -0.13 | -0.20 | -0.20 | 0.17 | 0.19 | -0.19 | -0.05 |
| **P53 status** | 0.04 | 0.01 | -0.12 | -0.03 | 0.06 | 0.07 | 0.15 | 0.42 | 1.00 | -0.24 | -0.30 | -0.27 | 0.06 | 0.21 | -0.05 | 0.02 |
| **Histological type** | -0.06 | -0.03 | 0.09 | 0.01 | -0.01 | -0.23 | 0.25 | -0.13 | -0.24 | 1.00 | 0.26 | 0.29 | 0.17 | 0.10 | -0.23 | 0.00 |
| **IDH1^+^, 1p/19q status** | -0.04 | -0.08 | 0.22 | 0.09 | -0.16 | 0.01 | -0.06 | -0.20 | -0.30 | 0.26 | 1.00 | 0.97 | 0.03 | -0.14 | 0.18 | 0.06 |
| **1p/19q status** | -0.01 | -0.05 | 0.22 | 0.11 | -0.16 | 0.01 | -0.06 | -0.20 | -0.27 | 0.29 | 0.97 | 1.00 | 0.03 | -0.10 | 0.15 | 0.06 |
| **MGMT status** | -0.01 | -0.10 | 0.02 | -0.05 | 0.00 | -0.17 | 0.26 | 0.17 | 0.06 | 0.17 | 0.03 | 0.03 | 1.00 | 0.07 | -0.10 | 0.08 |
| **Ki-67 index** | -0.05 | -0.08 | -0.09 | 0.05 | -0.06 | 0.33 | 0.18 | 0.19 | 0.21 | 0.10 | -0.14 | -0.10 | 0.07 | 1.00 | -0.17 | 0.00 |
| **Tumor volume** | 0.05 | 0.03 | 0.02 | -0.03 | 0.01 | 0.18 | -0.30 | -0.19 | -0.05 | -0.23 | 0.18 | 0.15 | -0.10 | -0.17 | 1.00 | 0.11 |
| **History of epilepsy** | 0.10 | 0.24 | 0.07 | 0.04 | -0.07 | 0.02 | -0.23 | -0.05 | 0.02 | 0.00 | 0.06 | 0.06 | 0.08 | 0.00 | 0.11 | 1.00 |

**Abbreviations: The best cut-off value of age, tumor volume was 47 years and 64.72 cm^3^, respectively. TC: Total Curvature; WHO: World Health Organization; IDH1: Isocitrate dehydrogenase 1; 1p/19q: chromosomal arms 1p and 19q; MGMT: O_6_-methylguanine-DNA methyltransferase; ATRX: Alpha thalassemia/mental retardation syndrome X-linked; TP53: Tumor protein p53; Ki-67: Ki-67 labeling index; IDH1^+^: IDH1 mutation**
