# Supplementary material for: The specific linear or curved boundaries between WHO grade II–III insular gliomas and the basal ganglia indicate distinct biological features, survival outcomes, and surgical strategies: evidence from 330 cases
Source: Neuroimage Clin. 2026 Apr 25;50:103995. doi: 10.1016/j.nicl.2026.103995 (PMC13141764; doi:10.1016/j.nicl.2026.103995)
Supplement: Supplementary Data 20 [file mmc20.docx]

**Supplement Table S12. The result of variance inflation factor analysis in C subgroup**

| **Variates** | **VIF** | **VIF condition** |
| --- | --- | --- |
| **TC** | 1.610210405 | Acceptable |
| **Tortuosity** | 1.712228765 | Acceptable |
| **Sex** | 1.153252908 | Acceptable |
| **Age** | 1.152102157 | Acceptable |
| **Side** | 1.126752165 | Acceptable |
| **WHO grade** | 1.46177155 | Acceptable |
| **IDH1 status** | 1.503659982 | Acceptable |
| **ATRX status** | 1.469029906 | Acceptable |
| **P53 status** | 1.384348294 | Acceptable |
| **Histological type** | 1.428501579 | Acceptable |
| **IDH1^+^, 1p/19q status** | 18.53061339 | Severe multicollinearity |
| **1p/19q status** | 18.26633691 | Severe multicollinearity |
| **MGMT status** | 1.171490914 | Acceptable |
| **Ki-67 index** | 1.331009366 | Acceptable |
| **Tumor volume** | 1.268454761 | Acceptable |
| **History of epilepsy** | 1.185731848 | Acceptable |

**Abbreviations: The best cut-off value of age, tumor volume was 47 years and 64.72 cm^3^, respectively. VIF: variance inflation factor; TC: Total Curvature; WHO: World Health Organization; IDH1: Isocitrate dehydrogenase 1; 1p/19q: chromosomal arms 1p and 19q; MGMT: O_6_-methylguanine-DNA methyltransferase; ATRX: Alpha thalassemia/mental retardation syndrome X-linked; TP53: Tumor protein p53; Ki-67: Ki-67 labeling index; IDH1^+^: IDH1 mutation**
