# Supplementary material for: The specific linear or curved boundaries between WHO grade II–III insular gliomas and the basal ganglia indicate distinct biological features, survival outcomes, and surgical strategies: evidence from 330 cases
Source: Neuroimage Clin. 2026 Apr 25;50:103995. doi: 10.1016/j.nicl.2026.103995 (PMC13141764; doi:10.1016/j.nicl.2026.103995)
Supplement: Supplementary Data 21 [file mmc21.docx]

**Supplement Table S13. The result of the stepwise Wald in C subgroup**

| **Covariate** | **beta** | **SE(beta)** | **HR** | **95%CI_lower** | **95%CI_upper** | **Wald_z** | **Wald_p** |
| --- | --- | --- | --- | --- | --- | --- | --- |
| **Ki-67 index** | 0.044413077 | 0.009813339 | 1.045414102 | 1.025498599 | 1.065716371 | 4.525786392 | 6.01713E-06 |
| **IDH1 status** | -0.858861504 | 0.407142325 | 0.423644125 | 0.190736867 | 0.940952568 | -2.109487152 | 0.034902554 |
| **Age** | 1.31266002 | 0.386205042 | 3.716045333 | 1.743157526 | 7.921827326 | 3.398868162 | 0.000676653 |
| **Tumor Volume** | 1.392330219 | 0.402007209 | 4.024216442 | 1.830146631 | 8.848645075 | 3.4634459 | 0.000533304 |
| **Tortuosity** | 1.192888078 | 0.402068196 | 3.296588276 | 1.499054256 | 7.249566995 | 2.966879971 | 0.003008384 |
| **1p/19q status** | -1.699593781 | 0.525036694 | 0.182757749 | 0.06530637 | 0.511441607 | -3.237095235 | 0.001207531 |

**Abbreviations: HR: Hazard Ratio; VIF: variance inflation factor; IDH1: Isocitrate dehydrogenase 1; 1p/19q: chromosomal arms 1p and 19q; TP53: Tumor protein p53; Ki-67: Ki-67 labeling index**
