# Supplementary material for: The specific linear or curved boundaries between WHO grade II–III insular gliomas and the basal ganglia indicate distinct biological features, survival outcomes, and surgical strategies: evidence from 330 cases
Source: Neuroimage Clin. 2026 Apr 25;50:103995. doi: 10.1016/j.nicl.2026.103995 (PMC13141764; doi:10.1016/j.nicl.2026.103995)
Supplement: Supplementary Data 23 [file mmc23.docx]

**Supplement Table S15. The matrix of Spearman's rank correlation analysis’ p value in GTR subgoup**

| **Variate** | **Sex** | **Age** | **Side** | **WHO grade** | **IDH1**  **status** | **ATRX**  **status** | **P53**  **status** | **Histological**  **type** | **IDH1^+^,**  **1p/19q status** | **1p/19q**  **status** | **MGMT**  **status** | **Ki-67**  **index** | **Tumor**  **volume** | **History of**  **epilepsy** | **Boundary**  **shape** |
| --- | --- | --- | --- | --- | --- | --- | --- | --- | --- | --- | --- | --- | --- | --- | --- |
| **Sex** | 0.000 | 0.359 | 0.465 | 0.945 | 0.437 | 0.041 | 0.912 | 0.057 | 0.025 | 0.036 | 0.796 | 0.621 | 0.140 | 0.030 | 0.266 |
| **Age** | 0.359 | 0.000 | 0.451 | 0.737 | 0.553 | 0.109 | 0.620 | 0.582 | 0.258 | 0.229 | 0.836 | 0.550 | 0.151 | 0.180 | 0.500 |
| **Side** | 0.465 | 0.451 | 0.000 | 0.215 | 0.893 | 0.965 | 0.970 | 0.576 | 0.412 | 0.080 | 0.506 | 0.350 | 0.444 | 0.754 | 0.150 |
| **WHO grade** | 0.945 | 0.737 | 0.215 | 0.000 | 0.920 | 0.348 | 0.008 | 0.139 | 0.447 | 0.367 | 0.011 | 0.254 | 0.138 | 0.767 | 0.329 |
| **IDH1 status** | 0.437 | 0.553 | 0.893 | 0.920 | 0.000 | 0.000 | 0.032 | 0.034 | 0.395 | 0.158 | 0.000 | 0.000 | 0.000 | 0.005 | 0.093 |
| **ATRX status** | 0.041 | 0.109 | 0.965 | 0.348 | 0.000 | 0.000 | 0.000 | 0.002 | 0.005 | 0.000 | 0.012 | 0.050 | 0.002 | 0.894 | 0.676 |
| **P53 status** | 0.912 | 0.620 | 0.970 | 0.008 | 0.032 | 0.000 | 0.000 | 0.000 | 0.000 | 0.000 | 0.157 | 0.528 | 0.465 | 0.936 | 0.005 |
| **Histological**  **type** | 0.057 | 0.582 | 0.576 | 0.139 | 0.034 | 0.002 | 0.000 | 0.000 | 0.000 | 0.000 | 0.001 | 0.000 | 0.035 | 0.894 | 0.000 |
| **IDH1^+^,**  **1p/19q status** | 0.025 | 0.258 | 0.412 | 0.447 | 0.395 | 0.005 | 0.000 | 0.000 | 0.000 | 0.000 | 0.490 | 0.004 | 0.591 | 0.848 | 0.000 |
| **1p/19q status** | 0.036 | 0.229 | 0.080 | 0.367 | 0.158 | 0.000 | 0.000 | 0.000 | 0.000 | 0.000 | 0.976 | 0.310 | 0.197 | 0.624 | 0.029 |
| **MGMT status** | 0.796 | 0.836 | 0.506 | 0.011 | 0.000 | 0.012 | 0.157 | 0.001 | 0.490 | 0.976 | 0.000 | 0.222 | 0.000 | 0.965 | 0.876 |
| **Ki-67 index** | 0.621 | 0.550 | 0.350 | 0.254 | 0.000 | 0.050 | 0.528 | 0.000 | 0.004 | 0.310 | 0.222 | 0.000 | 0.000 | 0.418 | 0.000 |
| **Tumor volume** | 0.140 | 0.151 | 0.444 | 0.138 | 0.000 | 0.002 | 0.465 | 0.035 | 0.591 | 0.197 | 0.000 | 0.000 | 0.000 | 0.475 | 0.051 |
| **History of**  **epilepsy** | 0.030 | 0.180 | 0.754 | 0.767 | 0.005 | 0.894 | 0.936 | 0.894 | 0.848 | 0.624 | 0.965 | 0.418 | 0.475 | 0.000 | 0.371 |
| **Boundary**  **shape** | 0.266 | 0.500 | 0.150 | 0.329 | 0.093 | 0.676 | 0.005 | 0.000 | 0.000 | 0.029 | 0.876 | 0.000 | 0.051 | 0.371 | 0.000 |

**Abbreviations: WHO: World Health Organization; IDH1: Isocitrate dehydrogenase 1; 1p/19q: chromosomal arms 1p and 19q; MGMT: O_6_-methylguanine-DNA methyltransferase; ATRX: Alpha thalassemia/mental retardation syndrome X-linked; TP53: Tumor protein p53; Ki-67: Ki-67 labeling index; IDH1^+^: IDH1 mutation**
