# Supplementary material for: The specific linear or curved boundaries between WHO grade II–III insular gliomas and the basal ganglia indicate distinct biological features, survival outcomes, and surgical strategies: evidence from 330 cases
Source: Neuroimage Clin. 2026 Apr 25;50:103995. doi: 10.1016/j.nicl.2026.103995 (PMC13141764; doi:10.1016/j.nicl.2026.103995)
Supplement: Supplementary Data 24 [file mmc24.docx]

**Supplement Table S16. The result of variance inflation factor analysis in GTR subgroup**

| **Variates** | **VIF** | **VIF condition** |
| --- | --- | --- |
| **Sex** | 1.163499823 | Acceptable |
| **Age** | 1.064843711 | Acceptable |
| **Side** | 1.06690975 | Acceptable |
| **WHO grade** | 1.196549847 | Acceptable |
| **IDH1 status** | 1.553320353 | Acceptable |
| **ATRX status** | 1.46263702 | Acceptable |
| **P53 status** | 1.534860387 | Acceptable |
| **Histological type** | 1.582262974 | Acceptable |
| **IDH1^+^, 1p/19q status** | 3.180280149 | Acceptable |
| **1p/19q status** | 3.026263672 | Acceptable |
| **MGMT status** | 1.299221848 | Acceptable |
| **Ki-67 index** | 1.596527684 | Acceptable |
| **Tumor volume** | 1.407048984 | Acceptable |
| **History of epilepsy** | 1.101778058 | Acceptable |
| **Boundary shape** | 1.739602942 | Acceptable |

**Abbreviations: VIF: variance inflation factor; WHO: World Health Organization; IDH1: Isocitrate dehydrogenase 1; 1p/19q: chromosomal arms 1p and 19q; MGMT: O_6_-methylguanine-DNA methyltransferase; ATRX: Alpha thalassemia/mental retardation syndrome X-linked; TP53: Tumor protein p53; Ki-67: Ki-67 labeling index; IDH1^+^: IDH1 mutation**
