# Supplementary material for: The specific linear or curved boundaries between WHO grade II–III insular gliomas and the basal ganglia indicate distinct biological features, survival outcomes, and surgical strategies: evidence from 330 cases
Source: Neuroimage Clin. 2026 Apr 25;50:103995. doi: 10.1016/j.nicl.2026.103995 (PMC13141764; doi:10.1016/j.nicl.2026.103995)
Supplement: Supplementary Data 26 [file mmc26.docx]

# Supplementary method protocol

1. Fractal dimension (FD)

**Formula:** $\text{FD}\text{ = }\lim_{\text{ε}\text{→}\text{0}} \frac{\log\text{N}\text{(}\text{ε}\text{)}}{\log\text{1}/\text{ε}}$

**Definition:** FD quantified the complexity or roughness of a curve by calculating how the number of segments $\text{N}\text{(}\text{ε}\text{)}$ needed to cover the curve changed as the scale $\text{ε}$ decreased.

**Explanation:** $\text{N}\text{(}\text{ε}\text{)}$: Number of segments required to cover the curve at scale $\text{ε}$.

$\log\text{1}/\text{ε}$: Logarithmic change in scale.

As $\varepsilon\to0$, the $\text{FD}\text{ = }\lim_{\text{ε}\text{→}\text{0}} \frac{\log\text{N}\text{(}\text{ε}\text{)}}{\log\text{1}/\text{ε}}$ reflected how the number of segments grown, capturing the curve's fractal nature and self-similarity.

Based on the mathematical concept of FD, two-dimension graphics' FD ≥ 2 (e.g. a circle); one-dimension graphics' FD ≤ 1 (e.g. a straight line). The FD of a curved line lied between 1 and 2.

1. Total curvature (TC)

**Formula:** $\text{Total}\text{ }\text{curvature}\text{ = }\int\left| \text{k}\text{(}\text{s}\text{)} \right|\text{ds}$

**Definition:** TC was defined as the integral of the absolute curvature along the entire curve, representing the overall bending or twisting of the curve.

**Explanation:** $\text{k}\text{(}\text{s}\text{)}$: Curvature at position $s$ along the curve, describing the local deviation from a straight line at each point.

$\mathrm{ds}$: Differential element of the arc length $\text{s}$, representing an infinitesimally small segment of the curve. Arc length $\text{ds}$ described the small segment between between two infinitesimally close points, and was used in integral calculus to describe the length of a curve.

TC was obtained by summing the absolute curvature over the entire curve. Larger values of TC indicated a curve with greater overall bending.

### Maximum curvature (MC)

****Formula**:** $\text{Maximum}\text{ }\text{curvature}\text{ = }\text{max}\text{ }\left| \text{k}\text{(}\text{s}\text{)} \right|$

#### **Definition:** MC referred to the highest curvature value at any point along the curve, which can help identify the sharpest bend.

#### **Explanation:** $\text{k}\text{(}\text{s}\text{)}$denotes the curvature at a given point on the curve, parameterized by arc length s. The maximum curvature was defined as the maximum absolute value of $\text{k}\text{(}\text{s}\text{)}$ along the entire curve.

MC indicated the sharpest bend in the curve, important for identifying points with extreme curvature.

1. Average curvature (AC)

**Formula:** $\text{Average}\text{ }\text{curvature}\text{ = }\frac{\text{1}}{\text{2}}\text{ (}\text{k}_{\text{1}}\text{+}\text{k}_{\text{2}}\text{)}$

**Definition:** AC provided an overall measure of how curved the curve was at a particular point by averaging the principal curvatures.

**Explanation:** $\text{k}_{\text{1}}$ and $\text{k}_{\text{2}}$: Principal curvatures at a point.

AC was the arithmetic mean of these two values, offering an average curvature that reflected the overall bending behavior of the curve at that point without extreme variations.

1. Curvature variation (CV)

**Formula:** $\text{Curvature}\text{ }\text{variation}\text{ = }\frac{\text{dκ}}{\text{d}\text{s}}$

**Definition:** CV measured the rate of change of curvature along the curve. It indicated how rapidly the bending of the curve changed as you moved along it.

**Explanation:** $\text{κ}$: Curvature at a point along the curve.

$\frac{\text{dκ}}{\text{d}\text{s}}$: Rate of change of curvature with respect to arc length $\text{s}$, showing how the curvature increased or decreased as you moved along the curve.

High CV values indicated significant changed in curvature, implying areas of the curve where bending was more variable.

1. Tortuosity

**Formula:** $\text{Tortuosity}\text{ = }\frac{\text{L}}{\text{D}}$

**Definition:** Tortuosity quantified the "winding" or "zigzag" nature of a curve. It was the ratio of the actual path length to the straight-line distance between two points.

**Explanation:** $\text{L}$: Actual length (arc length) of the curve.

$\text{D}$: Direct straight-line distance between the two endpoints of the curve.

Tortuosity described how much the curve deviated from a straight line. A tortuosity closed to 1 indicated a nearly straight line, while larger values indicated more winding or tortuous paths.
