# Supplementary material for: The specific linear or curved boundaries between WHO grade II–III insular gliomas and the basal ganglia indicate distinct biological features, survival outcomes, and surgical strategies: evidence from 330 cases
Source: Neuroimage Clin. 2026 Apr 25;50:103995. doi: 10.1016/j.nicl.2026.103995 (PMC13141764; doi:10.1016/j.nicl.2026.103995)
Supplement: Supplementary Data 27 [file mmc27.docx]

**Table S1. Detailed information on the pathological examination and antibodies**

| **Pathological examination** | | **Procedures** | **Antibodies details** | **Dilution** |
| --- | --- | --- | --- | --- |
| HE staining | | Obtained glioma tissues were fixed in 10% neutral formalin, embedded in paraffin, and sectioned at 3-5 μm. They were deparaffinized, rehydrated, stained with hematoxylin and eosin, dehydrated and mounted for microscopic examination. | - | - |
| IHC | IDH1 | Formalin-fixed, paraffin-embedded sections were processed as described for HE staining. The sections were incubated with primary antibodies, followed by horseradish peroxidase -conjugated secondary antibodies and visualization using DAB (3,3'-diaminobenzidine). Finally, sections were counterstained with hematoxylin, dehydrated through graded ethanol, cleared in xylene, and mounted with coverslips. | clone H09, Dianova, Hamburg, Germany | 1/60 |
|  | ATRX |  | ab97508, Abcam, Cambridge, UK | 1/800 |
|  | TP53 |  | clone BP53.12, Invitrogen, Waltham, USA | 1/200 |
|  | Ki–67 |  | clone MIB-1, Dako, Carpinteria, USA | 1/100 |
| FISH | 1p/19q co-delete | The pre-processed sections were treated to denature DNA. Locus-specific fluorescent probes targeting the 1p36 and 19q13 regions were applied, followed by overnight hybridization and stringent washing. The signals were then evaluated to determine the 1p/19q co-delete status. | Abbott Molecular, Illinois, USA | - |
| Pyrosequencing Analysis | MGMT | Genomic DNA was extracted from pre-processed sections and subjected to bisulfite conversion using a commercial kit. Subsequently, to amplify MGMT promoter region containing eight CpG sites in exon 1 on chromosome 10 and analyze the mean methylation level across the CpG sites. | Qiagen Hilden, Germany | - |

**Abbreviations:** HE: Hematoxylin–Eosin; IHC: Immunohistochemistry; FISH: Fluorescence in situ hybridization. IDH1: Isocitrate dehydrogenase 1; ATRX: Alpha thalassemia/mental retardation syndrome X-linked; TP53: Tumor protein p53; Ki–67: Ki–67 labeling index; 1p/19q co-delete: co-deletion of chromosomal arms 1p and 19q; MGMT: O_6_-methylguanine-DNA methyltransferase.
