# Supplementary material for: The specific linear or curved boundaries between WHO grade II–III insular gliomas and the basal ganglia indicate distinct biological features, survival outcomes, and surgical strategies: evidence from 330 cases
Source: Neuroimage Clin. 2026 Apr 25;50:103995. doi: 10.1016/j.nicl.2026.103995 (PMC13141764; doi:10.1016/j.nicl.2026.103995)
Supplement: Supplementary Data 29 [file mmc29.docx]

| **Environment** | **Details** |
| --- | --- |
| Hardware | The environment was based on Windows 11 (Microsoft Corporation, USA) system equipped with a 16-core CPU, 32 GB RAM, and an NVIDIA GeForce RTX 4050 Laptop GPU (6 GB VRAM). |
| Software | The environment was built on Python (version 3.10, Python Software Foundation, USA), and PyTorch (version 2025.1, JetBrains, Czech Republic). |
| Package | The necessary packages of model construction included numpy (version 2.1.3), pandas (version 2.2.3), matplotlib (version 3.10.1), lifelines (version 0.30.0), scipy (version 1.15.2), scikit-learn (version 1.6.1), openpyxl (version 3.1.5), and xlrd (version 2.0.2) |

**Table S3. Necessary infrastructure information for model construction**
