# Supplementary material for: The specific linear or curved boundaries between WHO grade II–III insular gliomas and the basal ganglia indicate distinct biological features, survival outcomes, and surgical strategies: evidence from 330 cases
Source: Neuroimage Clin. 2026 Apr 25;50:103995. doi: 10.1016/j.nicl.2026.103995 (PMC13141764; doi:10.1016/j.nicl.2026.103995)
Supplement: Supplementary Data 32 [file mmc32.docx]

**Table S6. The results of variance inflation factor analysis**

| **Variables** | **VIF** | **VIF condition** |
| --- | --- | --- |
| Gender | 1.065891758 | Acceptable |
| Age | 1.090039947 | Acceptable |
| Side | 1.156470812 | Acceptable |
| Tumor volume | 1.041770193 | Acceptable |
| History of epilepsy | 1.035446124 | Acceptable |
| Histological type | 1.419860422 | Acceptable |
| WHO grade | 1.164174681 | Acceptable |
| IDH1 status | 1.342535681 | Acceptable |
| 1p/19q status | 1.496722063 | Acceptable |
| IDH1**^+^**, 1p/19q status | 1.707371882 | Acceptable |
| MGMT status | 1.086311534 | Acceptable |
| ATRX status | 1.317228502 | Acceptable |
| TP53 status | 1.025770449 | Acceptable |
| Ki-67 index | 1.192526844 | Acceptable |

**Abbreviations:** VIF: Variance inflation factor; WHO: World Health Organization; IDH1: Isocitrate dehydrogenase 1; 1p/19q: chromosomal arms 1p and 19q; MGMT: O_6_-methylguanine-DNA methyltransferase; ATRX: Alpha thalassemia/mental retardation syndrome X-linked; TP53: Tumor protein p53; Ki-67: Ki-67 labeling index; IDH1**^+^**: IDH1 mutation. The best cut-off value of age, tumor volume was 38 years and 20.17 cm^3^, respectively.
