# Supplementary material for: The specific linear or curved boundaries between WHO grade II–III insular gliomas and the basal ganglia indicate distinct biological features, survival outcomes, and surgical strategies: evidence from 330 cases
Source: Neuroimage Clin. 2026 Apr 25;50:103995. doi: 10.1016/j.nicl.2026.103995 (PMC13141764; doi:10.1016/j.nicl.2026.103995)
Supplement: Supplementary Data 34 [file mmc34.docx]

**Table S8. The threshold values of specific continuous variables**

| **Group** | **Variables** | **Threshold value** | **No. of Patient** |
| --- | --- | --- | --- |
| L | Age (Years) | < 40 | 96 |
|  |  | ≥ 40 | 65 |
|  | Tumor volume (cm^3^) | < 71.79 | 82 |
|  |  | ≥ 71.79 | 79 |
| C | Age (Years) | < 40 | 67 |
|  |  | ≥ 40 | 102 |
|  | Tumor volume (cm^3^) | < 67 | 84 |
|  |  | ≥ 67 | 85 |
|  | Total Curvature | < 1.043 | 83 |
|  |  | ≥ 1.043 | 86 |
|  | Tortuosity | < 1.03 | 83 |
|  |  | ≥ 1.03 | 86 |

**Abbreviations:** L: linear; C: curved; No.: Number; The threshold value of age were identified by the clinical consensus. Other other threshold values were defined as the median because the data did not follow a normal distribution.
