# Supplementary material for: The specific linear or curved boundaries between WHO grade II–III insular gliomas and the basal ganglia indicate distinct biological features, survival outcomes, and surgical strategies: evidence from 330 cases
Source: Neuroimage Clin. 2026 Apr 25;50:103995. doi: 10.1016/j.nicl.2026.103995 (PMC13141764; doi:10.1016/j.nicl.2026.103995)
Supplement: Supplementary Data 35 [file mmc35.docx]

**Table S9. The matrix of Spearman's rank correlation coefficient analysis in the L subgroup**

| **Variables** | **Gender** | **Age** | **Side** | **WHO grade** | **IDH1 status** | **ATRX status** | **TP53**  **status** | **Histological**  **type** | **IDH1^+^,**  **1p/19q status** | **1p/19q**  **status** | **MGMT**  **status** | **Ki-67**  **index** | **Tumor**  **volume** | **History**  **of**  **epilepsy** |
| --- | --- | --- | --- | --- | --- | --- | --- | --- | --- | --- | --- | --- | --- | --- |
| Gender | 1.000 | 0.046 | 0.095 | 0.105 | 0.138 | -0.022 | 0.037 | 0.132 | 0.095 | 0.106 | -0.015 | 0.075 | -0.160 | 0.055 |
| Age | 0.046 | 1.000 | 0.084 | -0.088 | -0.049 | -0.159 | -0.123 | 0.068 | 0.178 | 0.128 | 0.044 | -0.015 | 0.053 | -0.118 |
| Side | 0.095 | 0.084 | 1.000 | -0.061 | 0.134 | 0.015 | -0.015 | -0.061 | 0.019 | -0.094 | 0.032 | 0.142 | 0.008 | -0.126 |
| WHO grade | 0.105 | -0.088 | -0.061 | 1.000 | 0.052 | 0.071 | 0.176 | -0.142 | 0.002 | -0.007 | -0.088 | 0.406 | 0.088 | 0.073 |
| IDH1 status | 0.138 | -0.049 | 0.134 | 0.052 | 1.000 | 0.343 | 0.171 | 0.128 | 0.267 | -0.160 | 0.198 | 0.242 | -0.425 | -0.179 |
| ATRX status | -0.022 | -0.159 | 0.015 | 0.071 | 0.343 | 1.000 | 0.467 | -0.215 | -0.185 | -0.306 | 0.109 | 0.112 | -0.142 | 0.005 |
| TP53 status | 0.037 | -0.123 | -0.015 | 0.176 | 0.171 | 0.467 | 1.000 | -0.330 | -0.216 | -0.275 | 0.082 | 0.082 | 0.023 | -0.023 |
| Histological  type | 0.132 | 0.068 | -0.061 | -0.142 | 0.128 | -0.215 | -0.330 | 1.000 | 0.330 | 0.293 | 0.191 | -0.061 | -0.054 | 0.013 |
| IDH1**^+^**,  1p/19q status | 0.095 | 0.178 | 0.019 | 0.002 | 0.267 | -0.185 | -0.216 | 0.330 | 1.000 | 0.498 | 0.121 | 0.115 | -0.119 | -0.017 |
| 1p/19q status | 0.106 | 0.128 | -0.094 | -0.007 | -0.160 | -0.306 | -0.275 | 0.293 | 0.498 | 1.000 | 0.067 | 0.018 | 0.112 | 0.055 |
| MGMT status | -0.015 | 0.044 | 0.032 | -0.088 | 0.198 | 0.109 | 0.082 | 0.191 | 0.121 | 0.067 | 1.000 | 0.075 | -0.219 | -0.018 |
| Ki-67 index | 0.075 | -0.015 | 0.142 | 0.406 | 0.242 | 0.112 | 0.082 | -0.061 | 0.115 | 0.018 | 0.075 | 1.000 | -0.150 | 0.000 |
| Tumor volume | -0.160 | 0.053 | 0.008 | 0.088 | -0.425 | -0.142 | 0.023 | -0.054 | -0.119 | 0.112 | -0.219 | -0.150 | 1.000 | 0.095 |
| History of epilepsy | 0.055 | -0.118 | -0.126 | 0.073 | -0.179 | 0.005 | -0.023 | 0.013 | -0.017 | 0.055 | -0.018 | 0.000 | 0.095 | 1.000 |

**Abbreviations:** WHO: World Health Organization; IDH1: Isocitrate dehydrogenase 1; ATRX: Alpha thalassemia/mental retardation syndrome X-linked; TP53: Tumor protein p53; 1p/19q: chromosomal arms 1p and 19q; MGMT: O_6_-methylguanine-DNA methyltransferase; Ki-67: Ki-67 labeling index; IDH1**^+^**: IDH1 mutation.
