# Supplementary material for: The specific linear or curved boundaries between WHO grade II–III insular gliomas and the basal ganglia indicate distinct biological features, survival outcomes, and surgical strategies: evidence from 330 cases
Source: Neuroimage Clin. 2026 Apr 25;50:103995. doi: 10.1016/j.nicl.2026.103995 (PMC13141764; doi:10.1016/j.nicl.2026.103995)
Supplement: Supplementary Data 36 [file mmc36.docx]

**Table S10. The matrix of p values of Spearman's rank correlation analysis in the L subgroup**

| **Variables** | **Gender** | **Age** | **Side** | **WHO grade** | **IDH1 status** | **ATRX status** | **TP53**  **status** | **Histological**  **type** | **IDH1+,**  **1p/19q**  **status** | **1p/19q**  **status** | **MGMT**  **status** | **Ki-67**  **index** | **Tumor**  **volume** | **History**  **of**  **epilepsy** |
| --- | --- | --- | --- | --- | --- | --- | --- | --- | --- | --- | --- | --- | --- | --- |
| Gender | 0.000 | 0.561 | 0.230 | 0.183 | 0.080 | 0.782 | 0.639 | 0.095 | 0.231 | 0.180 | 0.855 | 0.343 | 0.043 | 0.233 |
| Age | 0.561 | 0.000 | 0.290 | 0.269 | 0.537 | 0.044 | 0.121 | 0.393 | 0.024 | 0.105 | 0.584 | 0.849 | 0.502 | 0.338 |
| Side | 0.230 | 0.290 | 0.000 | 0.439 | 0.090 | 0.850 | 0.849 | 0.443 | 0.806 | 0.236 | 0.690 | 0.072 | 0.916 | 0.202 |
| WHO grade | 0.183 | 0.269 | 0.439 | 0.000 | 0.510 | 0.373 | 0.025 | 0.073 | 0.978 | 0.931 | 0.266 | 0.000 | 0.269 | 0.142 |
| IDH1 status | 0.080 | 0.537 | 0.090 | 0.510 | 0.000 | 0.000 | 0.030 | 0.106 | 0.001 | 0.043 | 0.012 | 0.002 | 0.000 | 0.118 |
| ATRX status | 0.782 | 0.044 | 0.850 | 0.373 | 0.000 | 0.000 | 0.000 | 0.006 | 0.019 | 0.000 | 0.170 | 0.159 | 0.073 | 0.997 |
| TP53 status | 0.639 | 0.121 | 0.849 | 0.025 | 0.030 | 0.000 | 0.000 | 0.000 | 0.006 | 0.000 | 0.301 | 0.302 | 0.770 | 0.423 |
| Histological  type | 0.095 | 0.393 | 0.443 | 0.073 | 0.106 | 0.006 | 0.000 | 0.000 | 0.000 | 0.000 | 0.015 | 0.439 | 0.493 | 0.126 |
| IDH1**+**,  1p/19q status | 0.231 | 0.024 | 0.806 | 0.978 | 0.001 | 0.019 | 0.006 | 0.000 | 0.000 | 0.000 | 0.126 | 0.147 | 0.133 | 0.649 |
| 1p/19q status | 0.180 | 0.105 | 0.236 | 0.931 | 0.043 | 0.000 | 0.000 | 0.000 | 0.000 | 0.000 | 0.399 | 0.820 | 0.156 | 0.854 |
| MGMT status | 0.855 | 0.584 | 0.690 | 0.266 | 0.012 | 0.170 | 0.301 | 0.015 | 0.126 | 0.399 | 0.000 | 0.347 | 0.005 | 0.633 |
| Ki-67 index | 0.343 | 0.849 | 0.072 | 0.000 | 0.002 | 0.159 | 0.302 | 0.439 | 0.147 | 0.820 | 0.347 | 0.000 | 0.057 | 0.580 |
| Tumor volume | 0.043 | 0.502 | 0.916 | 0.269 | 0.000 | 0.073 | 0.770 | 0.493 | 0.133 | 0.156 | 0.005 | 0.057 | 0.000 | 0.987 |
| History of epilepsy | 0.233 | 0.338 | 0.202 | 0.142 | 0.118 | 0.997 | 0.423 | 0.126 | 0.649 | 0.854 | 0.633 | 0.580 | 0.987 | 0.000 |

**Abbreviations:** WHO: World Health Organization; IDH1: Isocitrate dehydrogenase 1; ATRX: Alpha thalassemia/mental retardation syndrome X-linked; TP53: Tumor protein p53; 1p/19q: chromosomal arms 1p and 19q; MGMT: O_6_-methylguanine-DNA methyltransferase; Ki-67: Ki-67 labeling index; IDH1**^+^**: IDH1 mutation.
