# Supplementary material for: The specific linear or curved boundaries between WHO grade II–III insular gliomas and the basal ganglia indicate distinct biological features, survival outcomes, and surgical strategies: evidence from 330 cases
Source: Neuroimage Clin. 2026 Apr 25;50:103995. doi: 10.1016/j.nicl.2026.103995 (PMC13141764; doi:10.1016/j.nicl.2026.103995)
Supplement: Supplementary Data 37 [file mmc37.docx]

**Table S11. The results of the variance inflation factor analysis in the L subgroup**

| **Variables** | **VIF** | **VIF condition** |
| --- | --- | --- |
| Gender | 1.140682272 | Acceptable |
| Age | 1.100567244 | Acceptable |
| Side | 1.135413571 | Acceptable |
| WHO grade | 1.351181304 | Acceptable |
| IDH1 status | 1.854574033 | Acceptable |
| ATRX status | 1.53172838 | Acceptable |
| TP53 status | 1.515026091 | Acceptable |
| Histological type | 1.397529226 | Acceptable |
| IDH1+, 1p/19q status | 1.737043891 | Acceptable |
| 1p/19q status | 1.65115593 | Acceptable |
| MGMT status | 1.16527033 | Acceptable |
| Ki-67 index | 1.349155772 | Acceptable |
| Tumor volume | 1.385375878 | Acceptable |
| History of epilepsy | 1.092816945 | Acceptable |

**Abbreviations:** VIF: Variance inflation factor; WHO: World Health Organization; IDH1: Isocitrate dehydrogenase 1; ATRX: Alpha thalassemia/mental retardation syndrome X-linked; TP53: Tumor protein p53; 1p/19q: chromosomal arms 1p and 19q; MGMT: O6-methylguanine-DNA methyltransferase; Ki-67: Ki-67 labeling index; IDH1**^+^**: IDH1 mutation
