# Supplementary material for: The specific linear or curved boundaries between WHO grade II–III insular gliomas and the basal ganglia indicate distinct biological features, survival outcomes, and surgical strategies: evidence from 330 cases
Source: Neuroimage Clin. 2026 Apr 25;50:103995. doi: 10.1016/j.nicl.2026.103995 (PMC13141764; doi:10.1016/j.nicl.2026.103995)
Supplement: Supplementary Data 38 [file mmc38.docx]

**Table S12. The results of the stepwise Wald regression in the L subgroup**

| **Covariate** | **beta** | **SE(beta)** | **HR** | **95%CI_lower** | **95% CI_upper** | **Wald_z** | **Wald_p** |
| --- | --- | --- | --- | --- | --- | --- | --- |
| Ki-67 index | 0.78 | 0.31 | 2.19 | 1.19 | 4.02 | 2.52 | 0.01 |
| History of epilepsy | 0.54 | 0.32 | 1.72 | 0.93 | 3.20 | 1.72 | 0.09 |
| TP53 status | -0.77 | 0.32 | 0.46 | 0.25 | 0.88 | -2.37 | 0.02 |
| 1p/19q status | -0.90 | 0.46 | 0.40 | 0.16 | 1.00 | -1.96 | 0.05 |
| Tumor volume | 0.55 | 0.31 | 1.73 | 0.94 | 3.20 | 1.76 | 0.08 |

**Abbreviations:** HR: Hazard ratio; Ki-67: Ki-67 labeling index; TP53: Tumor protein p53; 1p/19q: chromosomal arms 1p and 19q; beta: Regression coefficient; SE (Beta): Standard error of the regression coefficient; HR: Hazard ratio; 95% CI Lower: 95% confidence interval lower bound; 95% CI Upper: 95% confidence interval upper bound; Wald_z: Wald statistic z value; Wald_p: Wald statistic p value
