# Supplementary material for: The specific linear or curved boundaries between WHO grade II–III insular gliomas and the basal ganglia indicate distinct biological features, survival outcomes, and surgical strategies: evidence from 330 cases
Source: Neuroimage Clin. 2026 Apr 25;50:103995. doi: 10.1016/j.nicl.2026.103995 (PMC13141764; doi:10.1016/j.nicl.2026.103995)
Supplement: Supplementary Data 39 [file mmc39.docx]

**Table S13. The corresponding points of the coefficient in the regression models**

| **Group** | **Covariate** | **beta** | **Points** |
| --- | --- | --- | --- |
| L | 1p/19q status | -0.90 | -100 |
|  | Ki-67 index | 0.78 | 87 |
|  | TP53 status | -0.77 | -85 |
|  | Tumor volume | 0.55 | 61 |
|  | History of epilepsy | 0.54 | 60 |
| C | 1p/19q status | -1.70 | -100 |
|  | Tumor volume | 1.39 | 82 |
|  | Age | 1.31 | 77 |
|  | Tortuosity | 1.19 | 70 |
|  | IDH1 status | -0.86 | -51 |
|  | Ki-67 index | 0.04 | 3 |

**Abbreviations:** L: linear; C: curved; 1p/19q: chromosomal arms 1p and 19q; Ki-67: Ki-67 labeling index; TP53: Tumor protein p53; IDH1: Isocitrate dehydrogenase 1.
