# Supplementary material for: The specific linear or curved boundaries between WHO grade II–III insular gliomas and the basal ganglia indicate distinct biological features, survival outcomes, and surgical strategies: evidence from 330 cases
Source: Neuroimage Clin. 2026 Apr 25;50:103995. doi: 10.1016/j.nicl.2026.103995 (PMC13141764; doi:10.1016/j.nicl.2026.103995)
Supplement: Supplementary Data 40 [file mmc40.docx]

**Table S14. The matrix of Spearman's rank correlation coefficient analysis in the C subgoup**

| **Variables** | **TC** | **Tortuosity** | **Gender** | **Age** | **Side** | **WHO**  **grade** | **IDH1**  **status** | **ATRX**  **status** | **TP53**  **status** | **Histological**  **type** | **IDH1^+^,**  **1p/19q**  **status** | **1p/19q**  **status** | **MGMT**  **status** | **Ki-67**  **index** | **Tumor volume** | **History of epilepsy** |
| --- | --- | --- | --- | --- | --- | --- | --- | --- | --- | --- | --- | --- | --- | --- | --- | --- |
| TC | 1.00 | 0.57 | -0.04 | 0.07 | -0.09 | 0.17 | -0.04 | 0.04 | 0.04 | -0.06 | -0.04 | -0.01 | -0.01 | -0.05 | 0.05 | 0.10 |
| Tortuosity | 0.57 | 1.00 | 0.05 | 0.08 | 0.07 | 0.07 | -0.12 | -0.05 | 0.01 | -0.03 | -0.08 | -0.05 | -0.10 | -0.08 | 0.03 | 0.24 |
| Gender | -0.04 | 0.05 | 1.00 | -0.02 | -0.03 | -0.02 | -0.18 | -0.20 | -0.12 | 0.09 | 0.22 | 0.22 | 0.02 | -0.09 | 0.02 | 0.07 |
| Age | 0.07 | 0.08 | -0.02 | 1.00 | -0.12 | 0.18 | -0.08 | -0.14 | -0.03 | 0.01 | 0.09 | 0.11 | -0.05 | 0.05 | -0.03 | 0.04 |
| Side | -0.09 | 0.07 | -0.03 | -0.12 | 1.00 | -0.12 | -0.08 | -0.05 | 0.06 | -0.01 | -0.16 | -0.16 | 0.00 | -0.06 | 0.01 | -0.07 |
| WHO grade | 0.17 | 0.07 | -0.02 | 0.18 | -0.12 | 1.00 | -0.18 | -0.10 | 0.07 | -0.23 | 0.01 | 0.01 | -0.17 | 0.33 | 0.18 | 0.02 |
| IDH1 status | -0.04 | -0.12 | -0.18 | -0.08 | -0.08 | -0.18 | 1.00 | 0.33 | 0.15 | 0.25 | -0.06 | -0.06 | 0.26 | 0.18 | -0.30 | -0.23 |
| ATRX status | 0.04 | -0.05 | -0.20 | -0.14 | -0.05 | -0.10 | 0.33 | 1.00 | 0.42 | -0.13 | -0.20 | -0.20 | 0.17 | 0.19 | -0.19 | -0.05 |
| TP53 status | 0.04 | 0.01 | -0.12 | -0.03 | 0.06 | 0.07 | 0.15 | 0.42 | 1.00 | -0.24 | -0.30 | -0.27 | 0.06 | 0.21 | -0.05 | 0.02 |
| Histological type | -0.06 | -0.03 | 0.09 | 0.01 | -0.01 | -0.23 | 0.25 | -0.13 | -0.24 | 1.00 | 0.26 | 0.29 | 0.17 | 0.10 | -0.23 | 0.00 |
| IDH1**^+^**, 1p/19q status | -0.04 | -0.08 | 0.22 | 0.09 | -0.16 | 0.01 | -0.06 | -0.20 | -0.30 | 0.26 | 1.00 | 0.97 | 0.03 | -0.14 | 0.18 | 0.06 |
| 1p/19q status | -0.01 | -0.05 | 0.22 | 0.11 | -0.16 | 0.01 | -0.06 | -0.20 | -0.27 | 0.29 | 0.97 | 1.00 | 0.03 | -0.10 | 0.15 | 0.06 |
| MGMT status | -0.01 | -0.10 | 0.02 | -0.05 | 0.00 | -0.17 | 0.26 | 0.17 | 0.06 | 0.17 | 0.03 | 0.03 | 1.00 | 0.07 | -0.10 | 0.08 |
| Ki-67 index | -0.05 | -0.08 | -0.09 | 0.05 | -0.06 | 0.33 | 0.18 | 0.19 | 0.21 | 0.10 | -0.14 | -0.10 | 0.07 | 1.00 | -0.17 | 0.00 |
| Tumor volume | 0.05 | 0.03 | 0.02 | -0.03 | 0.01 | 0.18 | -0.30 | -0.19 | -0.05 | -0.23 | 0.18 | 0.15 | -0.10 | -0.17 | 1.00 | 0.11 |
| History of epilepsy | 0.10 | 0.24 | 0.07 | 0.04 | -0.07 | 0.02 | -0.23 | -0.05 | 0.02 | 0.00 | 0.06 | 0.06 | 0.08 | 0.00 | 0.11 | 1.00 |

**Abbreviations:** TC: Total curvature; WHO: World Health Organization; IDH1: Isocitrate dehydrogenase 1; ATRX: Alpha thalassemia/mental retardation syndrome X-linked; TP53: Tumor protein p53; 1p/19q: chromosomal arms 1p and 19q; MGMT: O_6_-methylguanine-DNA methyltransferase; Ki-67: Ki-67 labeling index; IDH1**^+^**: IDH1 mutation.
