# Supplementary material for: The specific linear or curved boundaries between WHO grade II–III insular gliomas and the basal ganglia indicate distinct biological features, survival outcomes, and surgical strategies: evidence from 330 cases
Source: Neuroimage Clin. 2026 Apr 25;50:103995. doi: 10.1016/j.nicl.2026.103995 (PMC13141764; doi:10.1016/j.nicl.2026.103995)
Supplement: Supplementary Data 41 [file mmc41.docx]

**Table S15. The matrix of p values of Spearman's rank correlation analysis in the C subgoup**

| **Variables** | **TC** | **Tortuosity** | **Gender** | **Age** | **Side** | **WHO**  **grade** | **IDH1**  **status** | **ATRX**  **status** | **TP53**  **status** | **Histological**  **type** | **IDH1^+^,**  **1p/19q**  **status** | **1p/19q**  **status** | **MGMT**  **status** | **Ki-67**  **index** | **Tumor Volume** | **History of epilepsy** |
| --- | --- | --- | --- | --- | --- | --- | --- | --- | --- | --- | --- | --- | --- | --- | --- | --- |
| TC | 0.000 | 0.000 | 0.575 | 0.395 | 0.238 | 0.027 | 0.568 | 0.579 | 0.623 | 0.463 | 0.633 | 0.938 | 0.855 | 0.551 | 0.525 | 0.175 |
| Tortuosity | 0.000 | 0.000 | 0.540 | 0.279 | 0.337 | 0.336 | 0.124 | 0.514 | 0.908 | 0.673 | 0.293 | 0.500 | 0.198 | 0.320 | 0.684 | 0.002 |
| Gender | 0.575 | 0.540 | 0.000 | 0.843 | 0.656 | 0.818 | 0.017 | 0.009 | 0.107 | 0.256 | 0.005 | 0.005 | 0.778 | 0.253 | 0.832 | 0.401 |
| Age | 0.395 | 0.279 | 0.843 | 0.000 | 0.133 | 0.022 | 0.300 | 0.070 | 0.726 | 0.917 | 0.272 | 0.140 | 0.494 | 0.538 | 0.688 | 0.587 |
| Side | 0.238 | 0.337 | 0.656 | 0.133 | 0.000 | 0.125 | 0.321 | 0.521 | 0.434 | 0.850 | 0.041 | 0.041 | 0.977 | 0.467 | 0.849 | 0.359 |
| WHO grade | 0.027 | 0.336 | 0.818 | 0.022 | 0.125 | 0.000 | 0.017 | 0.215 | 0.343 | 0.003 | 0.862 | 0.862 | 0.023 | 0.000 | 0.023 | 0.798 |
| IDH1 status | 0.568 | 0.124 | 0.017 | 0.300 | 0.321 | 0.017 | 0.000 | 0.000 | 0.049 | 0.001 | 0.458 | 0.458 | 0.001 | 0.016 | 0.000 | 0.002 |
| ATRX status | 0.579 | 0.514 | 0.009 | 0.070 | 0.521 | 0.215 | 0.000 | 0.000 | 0.000 | 0.104 | 0.011 | 0.011 | 0.032 | 0.014 | 0.014 | 0.506 |
| TP53 status | 0.623 | 0.908 | 0.107 | 0.726 | 0.434 | 0.343 | 0.049 | 0.000 | 0.000 | 0.002 | 0.000 | 0.000 | 0.402 | 0.006 | 0.540 | 0.763 |
| Histological type | 0.463 | 0.673 | 0.256 | 0.917 | 0.850 | 0.003 | 0.001 | 0.104 | 0.002 | 0.000 | 0.001 | 0.000 | 0.024 | 0.204 | 0.003 | 0.978 |
| IDH1^+^, 1p/19q status | 0.633 | 0.293 | 0.005 | 0.272 | 0.041 | 0.862 | 0.458 | 0.011 | 0.000 | 0.001 | 0.000 | 0.000 | 0.652 | 0.075 | 0.019 | 0.426 |
| 1p/19q status | 0.938 | 0.500 | 0.005 | 0.140 | 0.041 | 0.862 | 0.458 | 0.011 | 0.000 | 0.000 | 0.000 | 0.000 | 0.652 | 0.204 | 0.049 | 0.426 |
| MGMT status | 0.855 | 0.198 | 0.778 | 0.494 | 0.977 | 0.023 | 0.001 | 0.032 | 0.402 | 0.024 | 0.652 | 0.652 | 0.000 | 0.333 | 0.188 | 0.287 |
| Ki-67 index | 0.551 | 0.320 | 0.253 | 0.538 | 0.467 | 0.000 | 0.016 | 0.014 | 0.006 | 0.204 | 0.075 | 0.204 | 0.333 | 0.000 | 0.027 | 0.957 |
| Tumor volume | 0.525 | 0.684 | 0.832 | 0.688 | 0.849 | 0.023 | 0.000 | 0.014 | 0.540 | 0.003 | 0.019 | 0.049 | 0.188 | 0.027 | 0.000 | 0.168 |
| History of epilepsy | 0.175 | 0.002 | 0.401 | 0.587 | 0.359 | 0.798 | 0.002 | 0.506 | 0.763 | 0.978 | 0.426 | 0.426 | 0.287 | 0.957 | 0.168 | 0.000 |

**Abbreviations:** TC: Total curvature; WHO: World Health Organization; IDH1: Isocitrate dehydrogenase 1; ATRX: Alpha thalassemia/mental retardation syndrome X-linked; TP53: Tumor protein p53; MGMT: O_6_-methylguanine-DNA methyltransferase; 1p/19q: chromosomal arms 1p and 19q; Ki-67: Ki-67 labeling index; IDH1^+^: IDH1 mutation.
