# Supplementary material for: The specific linear or curved boundaries between WHO grade II–III insular gliomas and the basal ganglia indicate distinct biological features, survival outcomes, and surgical strategies: evidence from 330 cases
Source: Neuroimage Clin. 2026 Apr 25;50:103995. doi: 10.1016/j.nicl.2026.103995 (PMC13141764; doi:10.1016/j.nicl.2026.103995)
Supplement: Supplementary Data 42 [file mmc42.docx]

**Table S16. The results of the variance inflation factor analysis in the C subgroup**

| **Variables** | **VIF** | **VIF condition** |
| --- | --- | --- |
| TC | 1.610210405 | Acceptable |
| Tortuosity | 1.712228765 | Acceptable |
| Gender | 1.153252908 | Acceptable |
| Age | 1.152102157 | Acceptable |
| Side | 1.126752165 | Acceptable |
| WHO grade | 1.46177155 | Acceptable |
| IDH1 status | 1.503659982 | Acceptable |
| ATRX status | 1.469029906 | Acceptable |
| TP53 status | 1.384348294 | Acceptable |
| Histological type | 1.428501579 | Acceptable |
| IDH1**^+^**, 1p/19q status | 18.53061339 | Severe multicollinearity |
| 1p/19q status | 18.26633691 | Severe multicollinearity |
| MGMT status | 1.171490914 | Acceptable |
| Ki-67 index | 1.331009366 | Acceptable |
| Tumor volume | 1.268454761 | Acceptable |
| History of epilepsy | 1.185731848 | Acceptable |

**Abbreviations:** VIF: Variance inflation factor; TC: Total Curvature; WHO: World Health Organization; IDH1: Isocitrate dehydrogenase 1; ATRX: Alpha thalassemia/mental retardation syndrome X-linked; TP53: Tumor protein p53; 1p/19q: chromosomal arms 1p and 19q; MGMT: O_6_-methylguanine-DNA methyltransferase; Ki-67: Ki-67 labeling index; IDH1**^+^**: IDH1 mutation.
