# Supplementary material for: The specific linear or curved boundaries between WHO grade II–III insular gliomas and the basal ganglia indicate distinct biological features, survival outcomes, and surgical strategies: evidence from 330 cases
Source: Neuroimage Clin. 2026 Apr 25;50:103995. doi: 10.1016/j.nicl.2026.103995 (PMC13141764; doi:10.1016/j.nicl.2026.103995)
Supplement: Supplementary Data 44 [file mmc44.docx]

| **Variables** | **Gender** | **Age** | **Side** | **WHO grade** | **IDH1**  **status** | **ATRX**  **status** | **TP53**  **status** | **Histological**  **type** | **IDH1^+^,**  **1p/19q status** | **1p/19q**  **status** | **MGMT**  **status** | **Ki-67** | **Tumor**  **volume** | **History of**  **epilepsy** | **Boundary**  **shape** |
| --- | --- | --- | --- | --- | --- | --- | --- | --- | --- | --- | --- | --- | --- | --- | --- |
| Gender | 1.000 | 0.067 | 0.053 | -0.005 | -0.057 | -0.149 | 0.008 | 0.138 | 0.162 | 0.152 | -0.019 | 0.036 | -0.108 | 0.157 | 0.081 |
| Age | 0.067 | 1.000 | 0.055 | 0.025 | -0.043 | -0.117 | 0.036 | -0.040 | 0.082 | 0.088 | -0.015 | -0.044 | 0.105 | -0.098 | -0.049 |
| Side | 0.053 | 0.055 | 1.000 | -0.090 | -0.010 | 0.003 | 0.003 | -0.041 | -0.060 | -0.127 | 0.048 | -0.068 | 0.056 | -0.023 | -0.105 |
| WHO grade | -0.005 | 0.025 | -0.090 | 1.000 | 0.007 | 0.069 | 0.191 | -0.108 | -0.056 | -0.066 | -0.185 | 0.083 | 0.108 | 0.022 | -0.071 |
| IDH1 status | -0.057 | -0.043 | -0.010 | 0.007 | 1.000 | 0.309 | 0.155 | 0.154 | 0.062 | -0.103 | 0.310 | 0.323 | -0.435 | -0.202 | 0.122 |
| ATRX status | -0.149 | -0.117 | 0.003 | 0.069 | 0.309 | 1.000 | 0.409 | -0.222 | -0.201 | -0.302 | 0.182 | 0.142 | -0.220 | -0.010 | -0.031 |
| TP53 status | 0.008 | 0.036 | 0.003 | 0.191 | 0.155 | 0.409 | 1.000 | -0.342 | -0.332 | -0.324 | 0.103 | -0.046 | -0.053 | 0.006 | -0.202 |
| Histological  type | 0.138 | -0.040 | -0.041 | -0.108 | 0.154 | -0.222 | -0.342 | 1.000 | 0.403 | 0.407 | 0.229 | 0.365 | -0.153 | -0.010 | 0.256 |
| IDH1**^+^**,  1p/19q status | 0.162 | 0.082 | -0.060 | -0.056 | 0.062 | -0.201 | -0.332 | 0.403 | 1.000 | 0.771 | 0.050 | 0.209 | 0.039 | -0.014 | 0.360 |
| 1p/19q status | 0.152 | 0.088 | -0.127 | -0.066 | -0.103 | -0.302 | -0.324 | 0.407 | 0.771 | 1.000 | 0.002 | 0.074 | 0.094 | 0.036 | 0.158 |
| MGMT status | -0.019 | -0.015 | 0.048 | -0.185 | 0.310 | 0.182 | 0.103 | 0.229 | 0.050 | 0.002 | 1.000 | 0.089 | -0.256 | -0.003 | -0.011 |
| Ki-67 | 0.036 | -0.044 | -0.068 | 0.083 | 0.323 | 0.142 | -0.046 | 0.365 | 0.209 | 0.074 | 0.089 | 1.000 | -0.324 | -0.059 | 0.704 |
| Tumor volume | -0.108 | 0.105 | 0.056 | 0.108 | -0.435 | -0.220 | -0.053 | -0.153 | 0.039 | 0.094 | -0.256 | -0.324 | 1.000 | 0.052 | -0.142 |
| History of  epilepsy | 0.157 | -0.098 | -0.023 | 0.022 | -0.202 | -0.010 | 0.006 | -0.010 | -0.014 | 0.036 | -0.003 | -0.059 | 0.052 | 1.000 | -0.065 |
| Boundary  shape | 0.081 | -0.049 | -0.105 | -0.071 | 0.122 | -0.031 | -0.202 | 0.256 | 0.360 | 0.158 | -0.011 | 0.704 | -0.142 | -0.065 | 1.000 |

**Table S18. The matrix of Spearman's rank correlation coefficient analysis in the GTR subgroup**

**Abbreviations:** WHO: World Health Organization; IDH1: Isocitrate dehydrogenase 1; ATRX: Alpha thalassemia/mental retardation syndrome X-linked; TP53: Tumor protein p53; 1p/19q: chromosomal arms 1p and 19q; MGMT: O_6_-methylguanine-DNA methyltransferase; Ki-67: Ki-67 labeling index; IDH1**^+^**: IDH1 mutation.
