# Supplementary material for: The specific linear or curved boundaries between WHO grade II–III insular gliomas and the basal ganglia indicate distinct biological features, survival outcomes, and surgical strategies: evidence from 330 cases
Source: Neuroimage Clin. 2026 Apr 25;50:103995. doi: 10.1016/j.nicl.2026.103995 (PMC13141764; doi:10.1016/j.nicl.2026.103995)
Supplement: Supplementary Data 46 [file mmc46.docx]

**Table S20. The results of the variance inflation factor analysis in the GTR subgroup**

| **Variables** | **VIF** | **VIF condition** |
| --- | --- | --- |
| Gender | 1.163499823 | Acceptable |
| Age | 1.064843711 | Acceptable |
| Side | 1.06690975 | Acceptable |
| WHO grade | 1.196549847 | Acceptable |
| IDH1 status | 1.553320353 | Acceptable |
| ATRX status | 1.46263702 | Acceptable |
| TP53 status | 1.534860387 | Acceptable |
| Histological type | 1.582262974 | Acceptable |
| IDH1**^+^**, 1p/19q status | 3.180280149 | Acceptable |
| 1p/19q status | 3.026263672 | Acceptable |
| MGMT status | 1.299221848 | Acceptable |
| Ki-67 | 1.596527684 | Acceptable |
| Tumor volume | 1.407048984 | Acceptable |
| History of epilepsy | 1.101778058 | Acceptable |
| Boundary shape | 1.739602942 | Acceptable |

**Abbreviations:** VIF: variance inflation factor; WHO: World Health Organization; IDH1: Isocitrate dehydrogenase 1; ATRX: Alpha thalassemia/mental retardation syndrome X-linked; TP53: Tumor protein p53; 1p/19q: chromosomal arms 1p and 19q; MGMT: O_6_-methylguanine-DNA methyltransferase; Ki-67: Ki-67 labeling index; IDH1**^+^**: IDH1 mutation
