# Supplementary material for: The specific linear or curved boundaries between WHO grade II–III insular gliomas and the basal ganglia indicate distinct biological features, survival outcomes, and surgical strategies: evidence from 330 cases
Source: Neuroimage Clin. 2026 Apr 25;50:103995. doi: 10.1016/j.nicl.2026.103995 (PMC13141764; doi:10.1016/j.nicl.2026.103995)
Supplement: Supplementary Data 47 [file mmc47.docx]

**Table S21. Multivariate regression analysis in the GTR subgroup**

| **Covariate** | **coef** | **exp(coef)** | **se(coef)** | **Coef**  **lower 95%** | **Coef**  **upper 95%** | **exp(coef)**  **lower 95%** | **exp(coef)**  **upper 95%** | ***p*** |
| --- | --- | --- | --- | --- | --- | --- | --- | --- |
| 1p/19q status | -1.38 | 0.25 | 0.58 | -2.52 | -0.24 | 0.08 | 0.79 | 0.02 |
| ATRX status | -0.25 | 0.78 | 0.40 | -1.03 | 0.52 | 0.36 | 1.69 | 0.52 |
| IDH1 status | -0.34 | 0.72 | 0.47 | -1.25 | 0.58 | 0.29 | 1.79 | 0.47 |
| Ki-67 index | 0.05 | 1.06 | 0.02 | 0.02 | 0.09 | 1.02 | 1.10 | 0.00 |
| MGMT status | -0.31 | 0.73 | 0.41 | -1.12 | 0.50 | 0.33 | 1.65 | 0.45 |
| TP53 status | -1.16 | 0.31 | 0.45 | -2.04 | -0.28 | 0.13 | 0.75 | 0.01 |
| WHO grade | 0.94 | 2.57 | 0.44 | 0.09 | 1.80 | 1.09 | 6.04 | 0.03 |
| Histological type | -0.09 | 0.91 | 0.45 | -0.97 | 0.78 | 0.38 | 2.19 | 0.84 |
| Side | -0.30 | 0.74 | 0.37 | -1.03 | 0.43 | 0.36 | 1.54 | 0.42 |
| History of epilepsy | 0.56 | 1.75 | 0.40 | -0.22 | 1.35 | 0.80 | 3.84 | 0.16 |
| Age | 0.11 | 1.11 | 0.38 | -0.64 | 0.86 | 0.53 | 2.36 | 0.78 |
| Boundary shape | -1.87 | 0.15 | 0.57 | -2.98 | -0.75 | 0.05 | 0.47 | 0.00 |
| Gender | 0.04 | 1.04 | 0.38 | -0.71 | 0.78 | 0.49 | 2.18 | 0.93 |
| Tumor Volume | 0.61 | 1.84 | 0.41 | -0.20 | 1.41 | 0.82 | 4.11 | 0.14 |

**Abbreviations:** GTR: gross total resection; 1p/19q: chromosomal arms 1p and 19q; ATRX: Alpha thalassemia/mental retardation syndrome X-linked; IDH1: Isocitrate dehydrogenase 1; Ki-67: Ki-67 labeling index; MGMT: O_6_-methylguanine-DNA methyltransferase; TP53: Tumor protein p53; WHO: World Health Organization; coef: Coefficient; exp(coef): Exponentiated coefficient; se(coef): Standard error of the coefficient; Coef lower 95%: Lower 95% confidence interval for the coefficient; Coef upper 95%: Upper 95% confidence interval for the coefficient; exp(coef) lower 95%: Lower 95% confidence interval for the exponentiated coefficient; exp(coef) upper 95%: Upper 95% confidence interval for the exponentiated coefficient; *p*: p value
